# A Multivariate Analysis of Genetic Constraints to Life History Evolution in a Wild Population of Red Deer

**DOI:** 10.1534/genetics.114.164319

**Published:** 2014-10-02

**Authors:** Craig A. Walling, Michael B. Morrissey, Katharina Foerster, Tim H. Clutton-Brock, Josephine M. Pemberton, Loeske E. B. Kruuk

**Affiliations:** * Institute of Evolutionary Biology, School of Biological Sciences, University of Edinburgh, Edinburgh, EH9 3JT, United Kingdom; † School of Biology, University of St. Andrews, St. Andrews, KY16 9TH, United Kingdom; ‡ Institute for Evolution and Ecology, University of Tübingen, Tübingen, D-72076 Germany; § Department of Zoology, University of Cambridge, Cambridge, CB2 3EJ, United Kingdom; ** Division of Evolution, Ecology, and Genetics, Research School of Biology, The Australian National University, Canberra, ACT 0200, Australia

**Keywords:** genetic correlations, life history trade-off, heritability, sexual antagonism, selection

## Abstract

Evolutionary theory predicts that genetic constraints should be widespread, but empirical support for their existence is surprisingly rare. Commonly applied univariate and bivariate approaches to detecting genetic constraints can underestimate their prevalence, with important aspects potentially tractable only within a multivariate framework. However, multivariate genetic analyses of data from natural populations are challenging because of modest sample sizes, incomplete pedigrees, and missing data. Here we present results from a study of a comprehensive set of life history traits (juvenile survival, age at first breeding, annual fecundity, and longevity) for both males and females in a wild, pedigreed, population of red deer (*Cervus elaphus*). We use factor analytic modeling of the genetic variance–covariance matrix (**G**) to reduce the dimensionality of the problem and take a multivariate approach to estimating genetic constraints. We consider a range of metrics designed to assess the effect of **G** on the deflection of a predicted response to selection away from the direction of fastest adaptation and on the evolvability of the traits. We found limited support for genetic constraint through genetic covariances between traits, both within sex and between sexes. We discuss these results with respect to other recent findings and to the problems of estimating these parameters for natural populations.

EVOLUTIONARY theory predicts low equilibrium genetic variation for fitness and fitness-related traits, because alleles that have negative effects on fitness should have been removed by selection, whereas those with positive effects should have reached fixation (Fisher 1958; Falconer 1981; Charlesworth 1987). The observation of strong selection on, and yet the persistence of genetic variation in, fitness-related traits when examined in isolation has led to the extension of this theory to multiple traits and the expectation of multivariate genetic constraints (Walsh and Blows 2009), where the majority of genetic variation segregating within a population should be the result of genes that have opposing effects on fitness through their effects on different traits (Falconer 1981; Lande 1982; Houle 1991; Roff 1996). Extension of theory to multiple traits has led to the prediction that, at equilibrium, further evolutionary change in traits under strong selection should be constrained or prohibited by genetic trade-offs—effectively, a lack of genetic variation in the direction of selection (Blows and Walsh 2009). However, despite their intuitive theoretical appeal, empirical support for these concepts is surprisingly scarce (Walsh and Blows 2009), especially for wild populations experiencing natural environments (Kruuk *et al.* 2008). Here, we use a multivariate framework to explore the role of genetic associations between life history traits in a wild population of red deer (*Cervus elaphus*) on the Isle of Rum, Northwest Scotland, and to consider in particular the relationship between traits expressed in either sex.

By far the most common approach to studying genetic trade-offs and genetic constraints is to estimate the bivariate genetic correlation between two traits (Blows and Hoffmann 2005; Walsh and Blows 2009). In particular, there has been a focus on the search for genetic correlations approaching −1 (or +1 if traits are selected in opposite directions) as these would represent an absolute constraint to bivariate trait evolution. However, given the inherently multivariate nature of selection and phenotypic variation, the focus on bivariate correlations may give a misleading impression of the extent of genetic constraints and in particular may lead to an underestimate of their importance (Dickerson 1955; Pease and Bull 1988). Indeed a mixture of positive and negative genetic correlations of intermediate magnitude can still result in limited or no genetic variation in the direction of selection when considering more than two traits (Walsh and Blows 2009). The focus on bivariate genetic correlations also ignores the importance of genetic variances as a potential source of genetic constraint (Agrawal and Stinchcombe 2009; Mcguigan and Blows 2010). Ultimately the degree to which multiple traits respond to selection is determined both by the distribution of genetic variances across those traits and by the genetic covariances among them, which jointly determine the amount of genetic variation that exists in the direction of selection (Lande 1979; Blows 2007). As such, it has been suggested that bivariate correlations are a poor indicator of genetic constraint and a more multivariate approach has been advocated (Dickerson 1955; Pease and Bull 1988; Walsh and Blows 2009; Houle *et al.* 2011).

As well as focusing on bivariate genetic correlations, the majority of previous studies have also focused on genetic correlations among traits expressed in the same sex as a potential cause of constraint (Roff 1996; Kruuk *et al.* 2008; Poissant *et al.* 2010). However, between-sex genetic correlations may also be important. For example, it has been hypothesized that because males and females often differ greatly in their reproductive roles and thus selective optima for different traits (Cox and Calsbeek 2009) and yet share the majority of their genome, there is the potential for sexually antagonistic genetic variation to exist, whereby genes that are beneficial to one sex are detrimental to the other (Lande 1980; Rice 1984; Bonduriansky and Chenoweth 2009). Evidence in support of sexually antagonistic genetic variation is accumulating from both laboratory (Chippindale *et al.* 2001; Fedorka and Mousseau 2004; Lewis *et al.* 2011) and natural populations (Brommer *et al.* 2007; Foerster *et al.* 2007; Mainguy *et al.* 2009; Cox and Calsbeek 2010).

Our aim in this study was to use multivariate techniques to assess the potential for genetic constraints to the evolution of four life history traits in a wild population of red deer (*C. elaphus*) on the Isle of Rum, Scotland. Previous studies in this population have shown genetic variation for numerous traits (Kruuk *et al.* 2000; Wilson *et al.* 2007; Nussey *et al.* 2008; Clements *et al.* 2011) and also, in line with theoretical predictions, that the heritability of traits decreases with increasing association with fitness [*i.e.*, increasing strength of selection (Kruuk *et al.* 2000)]. Life history traits in the Rum red deer population have lower heritabilities than morphological traits, but this is largely due to an increase in environmental variance for these traits (Kruuk *et al.* 2000). There is also evidence of sexually antagonistic genetic variation in the population (Foerster *et al.* 2007), with negative genetic correlations between estimates of male and female fitness, but the strength of this evidence differs slightly, depending on the measure of fitness used (see Foerster *et al.* 2007 and the associated supplementary information). More recently, Morrissey *et al.* (2012b) used a multivariate method proposed by Agrawal and Stinchcombe (2009) to provide evidence for genetic constraint through antagonistic correlations between female adult survival and female reproductive traits.

Here we extend the multivariate analysis of genetic constraint in the Rum red deer population to include both males and females and to study the effect of both genetic variances and within- and between-sex genetic covariances in generating constraint. We consider four life history traits, which together form a comprehensive set of all life history traits that determine individual fitness: survival to breeding age, age at first reproduction, longevity, and annual reproductive success. Our aims were split into two parts: (1) to quantify the genetic variance–covariance matrix (**G**) for females, males, and both sexes, with particular focus on characterizing the major multivariate axes of variation; and (2) to assess the degree of constraint imposed by the structure of **G** relative to the direction of selection using, first, estimates of the angle between the vector of selection and the vector of the predicted response (Smith and Rausher 2008) (“deflection” of the predicted response, *θ*) and, second, the length that the vector of the predicted response travels in the direction of selection [“evolvability,” *e*(***β***) (Hansen and Houle 2008)]. Part 2 necessarily involves characterization of the phenotypic process of selection on the different life history traits, which we undertake using a regression-based approach to estimate selection gradients. Although such estimates may not lead to robust predictions of evolutionary responses in situations when other, excluded, traits contribute to associations between trait and fitness (Rausher 1992; Morrissey *et al.* 2010), in an analysis of selection of complete life histories, all pathways by which effects of multivariate phenotype influence fitness are represented, because life history completely determines fitness. Thus there is by definition no unaccounted-for trait–fitness covariance in an analysis of complete life histories (we return to this point in the *Discussion*). Here, in particular, we assess estimates of deflection (*θ*) and evolvability [*e*(***β***)] when genetic covariances are fixed to zero *vs.* not zero, to assess the importance of genetic variance *vs.* covariances in generating any constraint.

## Materials and Methods

### General information

#### Study population:

We used individual life history information from red deer born between 1971 and 2007 in the study population in the North Block of the Isle of Rum, Inner Hebrides, Scotland (57° 03′ N, 06° 21′ W) (Clutton-Brock 1982). Individuals are recognizable from natural markings or artificial tags and data on life history traits are collected in weekly censuses conducted throughout the year and more intensive daily surveys during calving (May to July, when ∼50% of females give birth to a single calf) and mating (September to November) seasons (for details on data collection see Clutton-Brock 1982; Kruuk *et al.* 2002). Since 1982, ∼70% of calves have been caught soon after birth and artificially marked and an ear punch taken for genetic analysis. Other individuals have been sampled postmortem, from cast antlers, or by immobilization. All sampled individuals are genotyped at up to 15 microsatellite loci (Walling *et al.* 2010).

#### Pedigree determination:

Maternity was assigned with certainty based on observed associations between mothers and calves (Pemberton *et al.* 1988; Walling *et al.* 2010). Paternity was inferred from a combination of phenotypic and behavioral data (male age and the length of time a male held a female in his harem during her estrus window) and genetic data, using the paternity inference programs MasterBayes (Hadfield *et al.* 2006) and COLONY2 (Wang 2004; Wang and Santure 2009). Individual paternity assignments were accepted when their individual-level confidence was ≥80% (giving an average confidence in assignments of >98%). For paternal links, “dummy” sires were created to represent half-sibships between groups of offspring identified by COLONY to have a common (unsampled) father. For full details on paternity inference see Walling *et al.* (2010).

#### Life history traits studied:

We analyzed within- and between-sex variances and covariances in four life history traits that represent the major components of fitness in this population, measured separately in each sex to give a total of eight traits. The traits were as follows:

Survival to breeding age (SBA): Defined as 0 for all animals known to have died before and 1 for all animals known to have survived to May 1 in the year in which they reached the age of 3 years. Individuals whose fate was unknown or who died as a result of being shot outside the study area were removed from all analyses, resulting in a sample size of 1126 females (born to 462 mothers) and 1114 males (born to 437 mothers).
Age at first reproduction (AFR): The age in years at which a female first produced a calf or at which a male was first assigned paternity (*N* = 519 females and 149 males; many more males than females fail to breed). Because first breeding at an early age necessarily has a positive direct effect on fitness, AFR was multiplied by −1 in all multivariate analyses so that trade-offs would be represented by negative covariances and correlations.
Adult longevity (*L*): For both sexes, the age in years at death for individuals that survived to at least 3 years of age (*i.e.*, SBA = 1). Individuals that emigrated from the study area and thus for whom age at death was unknown were removed from the data set, as were individuals that died as a result of being shot outside the study area (121 females and 172 males in total). For both sexes, data were limited to individuals that were born before 1995 because for cohorts born from 1995 onward <80% of individuals were dead. This resulted in longevity values for 338 females and 245 males.
Annual breeding success (ABS): Defined as a repeated measure for all adults of breeding age. Females received a 0/1 score, with 1 representing years in which they produced a calf and 0 for years in which they did not. Females were included only if they were ≥3 years old and survived to at least 6 years of age; for each female ABS was recorded for each year until her death (or until 2008 if still living), so that several possible breeding attempts were included. This gave 3859 records from 439 individual females. For males, ABS was defined as the number of calves to which a male was assigned paternity for any given year in which males were ≥3 years old during the mating season (rut) and also were seen in the study area during the rut. Males were assigned an ABS score of 0 for a given year if they were seen in the study area during the corresponding rut, but not assigned paternity of any calves born the following year. Calves born in calendar year *x* are sired in the rut of calendar year *x −* 1. Thus male ABS is assigned to the year in which the calves were born rather than sired so that estimates of year-to-year variation in ABS in either sex correspond to the same calves. Paternity was assigned as described above and gave a total of 2004 records of ABS from 570 individual males.

For analyses of selection on these traits (see below), we also required estimates of individuals’ lifetime breeding success, defined as the total number of offspring produced across a lifetime, restricted to individuals known to have died a natural death.

To prevent differences in scale causing particular life history traits to dominate loadings in the factor analytic models described below, all traits were standardized to unit phenotypic variance by dividing by their standard deviation. In addition, AFR and male ABS were square-root transformed prior to standardization to better approximate normality.

A total of 1188 females were measured for at least one of the four life history traits under consideration in this study. The informative pedigree for these traits consisted of 1327 maternal links (including mothers that lacked phenotypic information but were informative for relatedness) and 893 paternal links. For males a total of 1369 individuals were measured for at least one trait and the informative pedigree consisted of 1586 maternal links and 1077 paternal links (of which 141 were dummy sires). When combining data on both sexes, 2557 individuals were scored for at least one trait, with the informative pedigree consisting of 2368 maternal links and 1573 paternal links. For all analyses, the pedigree had a maximum depth of eight generations with a mode of three generations of information for each individual.

### Part 1: Variance decomposition: Estimating G

#### Univariate analysis:

For each trait, variance components and appropriate random effects structures were initially investigated using univariate animal models of the general form

y=μ+Xb+Zu+e, (1)

where **y** is a vector of phenotypic observations, *μ* is the mean, **b** is a vector of fixed effects, **u** is a vector of random effects, **X** and **Z** are design matrices linking individual records to the appropriate fixed and random effects, and **e** is a vector of residual errors.

Animal models are a form of linear mixed-effect model that use pedigree information to decompose phenotypic variance into components due to additive genetic and other effects (Henderson 1976; Kruuk 2004). The random effects fitted (and thus variance components estimated) differed between traits: additive genetic (*V*_A_), year of birth (*V*_BY_), and residual (*V*_R_) effects were modeled for all traits; maternal effects [*V*_M_—the influence of a mother’s phenotype on that of her offspring, independent of additive genetic effects (Kruuk and Hadfield 2007)] were modeled for early life traits SBA and AFR; and permanent environment [*V*_PE_—constant environmental influences on an individual’s phenotype across repeated measures on that individual (Kruuk and Hadfield 2007)] and year of measure (*V*_YR_) effects for ABS were modeled because of its repeated measures on individuals across multiple years. Birth year and year of measurement were included to test for between-cohort and between-year environmental variation, respectively, such as that due to population density and climate variables (Kruuk *et al.* 2002). The statistical significance of random effects was tested by comparing full models to models excluding specific random effects, using likelihood-ratio tests, with twice the difference in log-likelihood being *χ*^2^ distributed with 1 d.f. for every additional parameter fitted. Nonsignificant random effects, apart from additive genetic effects, were removed from final models. Fixed effects previously shown to be important in this system were also included and are detailed in Supporting Information, File S1.

### Multivariate analysis

#### Phenotypic covariation:

To estimate within-sex phenotypic covariances among traits, we ran multivariate equivalents of the models represented by Equation 1, where **y** now represents a matrix of phenotypic observations of all traits measured within each sex and *μ* is a vector of means for each phenotypic trait. SBA was not included in these models because only individuals that score 1 for SBA can have a phenotypic value for any other trait and thus the phenotypic covariance between SBA and other traits is undefined. These models contained fixed effects as described in File S1, but only a single, individual-level, random effect defining individual-level variance (*V*_I_, equivalent to individual repeatability) for all traits. By fixing the residual variance for single-measures traits (AFR and *L*) to zero, this model structure allows the residual (after correcting for fixed effects) variance for these traits to be represented by the individual-level variance, allowing estimation of the phenotypic covariance between AFR, *L*, and the individual-level repeatability of ABS (Morrissey *et al.* 2012a). This does not imply that we can estimate the repeatable component of variation for traits (AFR and *L*) that are measured only once; rather, the model structure allows estimation of the biologically interesting phenotypic relationship between AFR, *L*, and the repeatable component of ABS, which is the phenotypic covariance between these traits (Morrissey *et al.* 2012a). Between-sex phenotypic covariances are undefined and thus set to zero, as no sex ever expresses the phenotype of the opposite sex.

#### Genetic (co)variation:

We estimated **G** matrices for females (**G_f_**) and males (**G_m_**) separately and then for both sexes together (**G_bs_**). To do this, we again ran full multivariate equivalents of the model represented in Equation 1 for each sex and then both sexes combined, but this time included the additive genetic and all significant random effects identified in the univariate models (above). Covariances were estimated between all variance components where they were definable. As in models of phenotypic covariances, residual variances for singly measured traits (AFR and *L*) were fixed to zero, allowing estimation of the covariance between residual and nongenetic permanent environment variances of single- and repeated-measures traits, respectively. Nongenetic random effects were modeled as variance-correlation matrices with correlations constrained to be positive definite (*i.e.*, bounded by ±1) as these proved more stable and gave parameter estimates that were within the realms of possibility (Gilmour *et al.* 2009). The significance of correlations was assessed using likelihood-ratio tests as above, comparing models where correlations were estimated to those where the correlation was fixed at 0.

To overcome issues associated with the large number of parameters to be estimated in a multivariate **G** matrix (see File S1) we used factor analytic modeling (factor analysis, FA) techniques (Wright 1932). We discuss these methods in detail in File S1, but give a brief overview here. We first constructed sex-specific models of the four life history traits in either sex and then considered both sexes together. Specifically we modeled the genetic variance–covariance matrix (**G**) as a product of a number *m* of independent linear combinations of the original (*p*) traits,

$$\hat{G}=\Lambda \Lambda^{T},$$ (2)

where $\hat{G}$ is a (potentially reduced-rank) estimate of **G**, Λ is a lower triangle matrix of constants that represent loadings of each trait on each factor, and ^T^ is the transpose of a matrix. FA can be performed in ASReml (Thompson *et al.* 2003; Gilmour *et al.* 2009) and the significance of additional factors can be assessed by comparing the log-likelihoods of models with sequentially more (or fewer) factors. Twice the difference between the log-likelihoods of successive models was assumed to be chi-square distributed with d.f. equal to the change in d.f. between models in statistical hypothesis tests. A full-rank FA model, with Λ representing a lower triangle of a matrix of dimension equal to the number of traits (for Equation 2), provides a multivariate estimate of **G**, with identical values and associated likelihood to an unconstrained estimate.

Although FA has been used to assess the rank of **G** (*e.g.*, Mezey and Houle 2005; Hine and Blows 2006), doing so may result in an underestimate of the rank of **G** (Hill and Thompson 1978; Meyer and Kirkpatrick 2008; see File S1 for more details). Here, we took an alternative approach of “building up” an FA model, adding additional factors until either **G** was full rank {rank Λ = *p* [four (within-sex models) or eight (both-sex models) in this case]} or it was not possible to add additional factors to a model (due to failure of convergence). FA modeling allows estimation of $\hat{G}$ (*i.e.*, ΛΛ^T^) that contains the maximum possible variance estimable given the data and thus provides the best possible estimate of **G** with which to subsequently assess its potential to generate evolutionary constraint (see below). Because the leading factors to be estimated are those that contain the most variance, any unestimable factors in our analysis should explain considerably less variance than those that have already been estimated and would thus contribute much less to a predicted response to selection than those that are included.

### Part 2: Assessing the potential for genetic constraint to evolution

The majority of methods for estimating multivariate genetic constraint center around Lande’s (1979) formulation of the multivariate breeders’ equation

$$\Delta \bar{z}=G\beta ,$$ (3)

where $\Delta \bar{z}$ is a vector of predicted changes in trait means over a single generation, **G** is the multivariate genetic variance–covariance matrix, and **β** is the vector of directional selection gradients (Lande 1979; Lande and Arnold 1983). When considering traits expressed in each sex, this becomes

$$\Delta \bar{z}=\frac{1}{2}\left(\begin{array}{cc} G_{f} & B\\ B^{T} & G_{m} \end{array}\right)\left(\begin{array}{c} \beta_{f}\\ \beta_{m} \end{array}\right)$$ (4)

(Lande 1980), where **G_f_** is the additive genetic (co)variance matrix for females, **G_m_** is the additive genetic (co)variance matrix for males, **B** is the genetic covariance matrix between the sexes, **^T^** is the transpose of a matrix, **β_m_** is the vector of selection gradients for male traits, and **β_f_** is the vector of selection gradients for female traits (Lande 1980). The factor 1/2 is required because male and female parents make equal autosomal contributions to the offspring of both sexes. Equation 4 demonstrates that the predicted response to selection ($\Delta \bar{z}$) is scaled and deflected away from the direction of maximal adaptation (*i.e.*, the direction in which population mean fitness increases most rapidly as a function of phenotype), as defined by the vector of selection gradients (**β**), by **G**. The degree of genetic constraint can therefore be summarized by comparing the vector of the predicted response to selection ($\Delta \bar{z}$) to the vector of selection itself (**β**). Comparison of vectors can be done in two related ways (details below); doing so first required calculation of selection gradients for the traits under study.

### Calculating selection gradients (β)

We initially calculated selection *differentials* (**S**) for all traits in this study from the covariance between the trait and relative fitness (Lande and Arnold 1983). Traits were standardized as for genetic analyses and the same fixed-effects structures were fitted to the regression models as to the animal models above. Because individuals have to survive to breeding age (*i.e.*, score SBA = 1) to score for all other traits (AFR, *L*, and ABS), we analyzed selection as a two-step process, analyzing selection on SBA separately from selection on AFR, *L*, and ABS. Relative fitness for selection on SBA was defined as the ratio of lifetime breeding success (the total number of offspring produced) of an individual to the sex-specific population mean lifetime breeding success of individuals with known SBA. Relative fitness for selection on all other traits (AFR, *L*, and ABS) was defined as the ratio of lifetime breeding success of an individual to the sex-specific population mean lifetime breeding success of individuals that survived to breeding age (*i.e.*, had an SBA score of 1) and had a known phenotype for at least one of AFR, *L*, and ABS.

To calculate **S** for each trait, bivariate models of the form in Equation 1 were fitted with both relative fitness and the trait of interest (*i.e.*, female SBA, male SBA, female AFR, etc.) as dependent variables. For the single-measures traits (SBA, AFR, and *L*), no random effects (other than residual) were fitted and the selection differentials were estimated as the phenotypic covariance between the standardized trait and relative fitness. For the repeated-measures trait ABS, the relationship with fitness was calculated by fitting a bivariate model of ABS and fitness with an individual-level term for both traits, similar to the models used above. The residual variance for fitness was then fixed at zero, forcing the residual variance for fitness to be represented by the individual-level term and thus allowing the estimation of the phenotypic covariance between relative fitness and the individual repeatability of ABS (similar to that above; see also Morrissey *et al.* 2012a). Sample sizes for these models were 757 for female SBA, 262 for female AFR, 278 for female *L*, 254 individuals with 2479 observations for female ABS, 723 for male SBA, 81 for male AFR, 121 for male *L*, and 127 individuals with 849 measures of ABS.

Sex-specific selection *gradients* (**β**) were then calculated from the equation

$$\beta =P^{-1}S,$$ (5)

where **β** is a vector of selection gradients, **P** is the phenotypic variance–covariance matrix for each sex (calculated as above, Table S2), and **S** is a vector of selection differentials (Lande and Arnold 1983). The phenotypic covariance between SBA and all other traits is undefined because only individuals that score 1 for SBA can score for any other trait. Thus standardized selection gradients for SBA are equal to the selection differential. Results from this approach are similar to results removing SBA from all analyses (data not shown), apart from the specific case of female evolvability (see *Discussion* in File S1). We calculated three vectors of selection gradients: a vector of selection gradients on female traits (**β_f_**); a vector of selection gradients on male traits (**β_m_**); and then, by combining **β_f_** and **β_m_**, a vector of selection gradients for both female and male traits (**β_bs_**). Standard errors for **S** and **P** are estimated within ASReml.

### Metrics of constraint

#### Deflection (*θ*), the angle between the predicted response to selection ($\Delta \bar{z}$) and the selection gradient (β):

To assess the strength of genetic constraints to evolution we calculated the angle (*θ*) between the vector of selection (**β**) and the predicted response to selection ($\Delta \bar{z}$) (Smith and Rausher 2008) as

$$\theta ={\text{cos}}^{-\text{1}}\left(\frac{\Sigma (\Delta z\beta )}{\sqrt{\Sigma \Delta z}\sqrt{\Sigma \beta }}\right)\frac{\mathrm{180}}{\pi }.$$ (6)

*θ* provides an estimate of the degree to which **G** deflects evolutionary trajectories away from the direction of selection and is thus a representation of the degree of genetic constraint. The use of angles between vectors can be extended (Agrawal and Stinchcombe 2009) to assess the influence of genetic covariances on the response to selection by calculating the predicted response to selection ($\Delta \bar{z}$**_nc_**) when *all* covariances within **G** are set to zero (**G_nc_**, where _nc_ = no covariances). Different angles can then be calculated to assess the effect of various aspects of **G** on the predicted response to selection ($\Delta \bar{z}$), with larger angles suggesting an increase in constraint. We calculated four angles for within- and both-sex analyses: *θ*_1_, the angle between **β_(f, m, or bs)_** and $\Delta \bar{z}$ **_(f, m, or bs)_**, the combined effect of unequal genetic variances and nonzero covariances on deflection; *θ*_2_, the angle between **β_(f, m, and bs)_** and $\Delta \bar{z}$ **_(f, m, and bs)nc_**, the extent to which unequal variances cause deflection; *θ*_3_, the angle between $\Delta \bar{z}$ **_(f, m, or bs)_** and $\Delta \bar{z}$ **_(f, m, and bs)nc_**, the effect of nonzero covariances on the direction of the predicted response to selection; and *θ*_4_, the difference between *θ*_1_ and *θ*_2_ (*θ*_1_ − *θ*_2_), the amount that genetic covariances alter deflection (positive values indicate covariances increase constraint).

For the both-sex analysis, we calculated three additional angles: *θ*_5_bs_, the angle between **β_bs_** and $\Delta \bar{z}$ when just the between-sex covariances are set to zero (*i.e.*, using **G_nbs_** in Equation 4 to calculate $\Delta \bar{z}$**_nbs_**, where _nbs_ = no between-sex covariances), which represents the combined effect of unequal genetic variances and within-sex covariances on deflection; *θ*_6_bs_, the angle between $\Delta \bar{z}$**_bs_** and $\Delta \bar{z}$**_nbs_**, the effect of nonzero between-sex covariances on the direction of the predicted response to selection; and *θ*_7_bs_, the difference between *θ*_1_bs_ and *θ*_5_bs_ (*θ*_1_bs_ − *θ*_5_bs_), the amount that between-sex covariances alter deflection (positive values indicate increased constraint, see Table 2).

#### Evolvability *e*(β):

**Figure 1:**
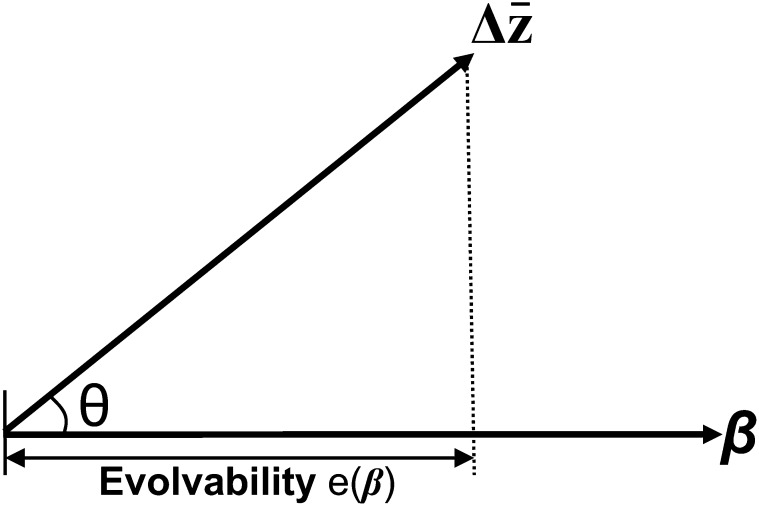
Two-dimensional illustration of deflection (*θ*) and evolvability [*e*(**β**)], the measures of constraint. *θ* is the angle between the vector of selection (**β**) and the predicted response to selection ($\Delta \bar{z}$). Evolvability *e*(**β**) is the length of the projection of $\Delta \bar{z}$ onto **β**, as a proportion of the length of **β** (Equation 7), and represents the magnitude of the predicted response to selection in the direction of selection; adapted from Hansen and Houle (2008).

Although *θ* provides a good measure of the degree to which $\Delta \bar{z}$ is deflected away from **β** by **G**, it does not take into account any effect of **G** on the magnitude of the responses. Thus another way of assessing constraint is to calculate Hansen and Houle’s (2008) evolvability metric [*e*(**β**)] (Figure 1) defined as

$$e(\beta )=\frac{\beta^{\text{T}}G\beta }{{\left|\beta \right|}^{2}},$$ (7)

where ***β***, **G**, and ^T^ are as defined above and **||** is the norm of the vector.

Evolvability corresponds to the length of the projection of $\Delta \bar{z}$ onto **β** and thus describes the length of the response in the direction of selection (Figure 1) and is standardized by the strength of selection (*i.e.*, is given as a proportion of the length (norm) of the vector of selection (|**β**|^2^ in Equation 7). It summarizes the effect of **G** on $\Delta \bar{z}$ in terms of both deflecting $\Delta \bar{z}$ away from the direction of **β** and adjusting the magnitude of the response. For comparison with evolvability, a useful benchmark is the average evolvability (*ē*) over random selection gradients, defined as

$$\bar{e}=\frac{\sum_{i}\lambda_{i}}{k},$$ (8)

where *λ_i_* are eigenvalues of **G** and the sum is over all *k* eignevalues (Hansen and Houle 2008). This is equivalent to the average additive genetic variance of the traits and thus provides a measure of the evolutionary potential of **G** independent of the strength and direction of selection (Hansen and Houle 2008; Innocenti and Chenoweth 2013). Calculating this average evolvability over random selection gradients for females ($\bar{e}$_f_), males ($\bar{e}$_m_), and both sexes ($\bar{e}$_bs_) gives 0.111, 0.224, and 0.181, respectively.

As with deflection, we calculated a number of values of evolvability. For female, male, and both-sex models, we calculated the evolvability using **G_(f, m, and bs)_**, where both genetic variances and covariances were estimated [evolvability *e*(**β_f_**), *e*(**β_m_**), and *e*(**β_bs_**)]. *e*(**β_f_**), *e*(**β_m_**), and *e*(**β_bs_**) provide an estimate of the effect of both genetic variances and covariances on evolvability. We also calculated evolvability fixing genetic covariances to 0 [*i.e.*, using **G_(f, m and bs)nc_**], providing an estimate of the evolvability based on the genetic variances alone [evolvability *e*(**β_f_**)_nc_, *e*(**β_m_**)_nc_, and *e*(**β_bs_**)_nc_]. *e*(**β_x_**) and *e*(**β_x_**)_nc_ can then be compared to provide an estimate of the degree to which genetic covariances alter evolvability,

$$R_{\text{e}\_x}=\frac{e\left(\beta_{x}\right)}{e{\left(\beta_{x}\right)}_{\mathrm{nc}}},$$ (9)

where *x* = *f*, *m*, or *bs* (Morrissey *et al.* 2012b). Thus an *R*_e_*__x_* value <1 suggests that genetic covariances reduce the predicted evolvability compared to the effect of genetic variances and increase constraint (Morrissey *et al.* 2012b).

For both-sex models we also calculated evolvability fixing between sex covariances to zero [**G_(bs)nbs_** giving evolvability *e*(**β_bs_**)_nbs_]. *e*(**β_bs_**)_nbs_ allowed calculation of two additional values of *R*_e_: (1) *R*_e_bs_nbs_ = *e*(**β_bs_**)/*e*(**β_bs_**)_nbs_ and (2) *R*_e_bs_nbs.nc_ = *e*(**β_bs_**)_nbs_ /*e*(**β_bs_**)_nc_. *R*_e_bs_nbs_ compares evolvability with and without between-sex genetic covariances fixed to 0, with a value of less than one indicating that between-sex genetic covariances reduce predicted evolvability and thus increase the constraint. *R*_e_bs_nbs.nc_ compares evolvability with and without within-sex genetic covariances fixed to 0, with a value of less than one indicating that within-sex genetic covariances reduce the predicted evolvability and thus increase constraint.

All models were run in ASReml version 3.0 (Gilmour *et al.* 2009). The restricted maximum-likelihood procedures used here assume that residuals are normally distributed, having conditioned on the fixed and random effects structures, although they are likely to be fairly robust to departures from these assumptions (Lynch and Walsh 1998, p. 784). Even so, SBA and female ABS are binary traits. As such, estimates of the heritability of these traits based on observed data may be underestimates compared to estimates based on an underlying liability scale (Lynch and Walsh 1998; Roff 2001). However, because this underestimate of the variance also affects any estimate of the covariance, estimates of the genetic correlations should be unbiased (Brotherstone *et al.* 1990; Lynch and Walsh 1998; Roff 2001). Although methods exist that allow appropriate error structures for each trait to be fitted (Hadfield 2010), these methods do not currently allow FA models to be fitted and so could not be used here. In addition, a generalized model would generate estimates of (co)variance components on a latent scale that would not readily be combined with estimates of selection gradients to predict the response to selection ($\Delta \bar{z}$). As such, we present estimates from models assuming normally distributed residuals throughout. Simulation-based credible intervals (see below) and the comparison of vectors were performed in R version 2.12.0 (R Development Core Team 2010).

**Figure 2:**
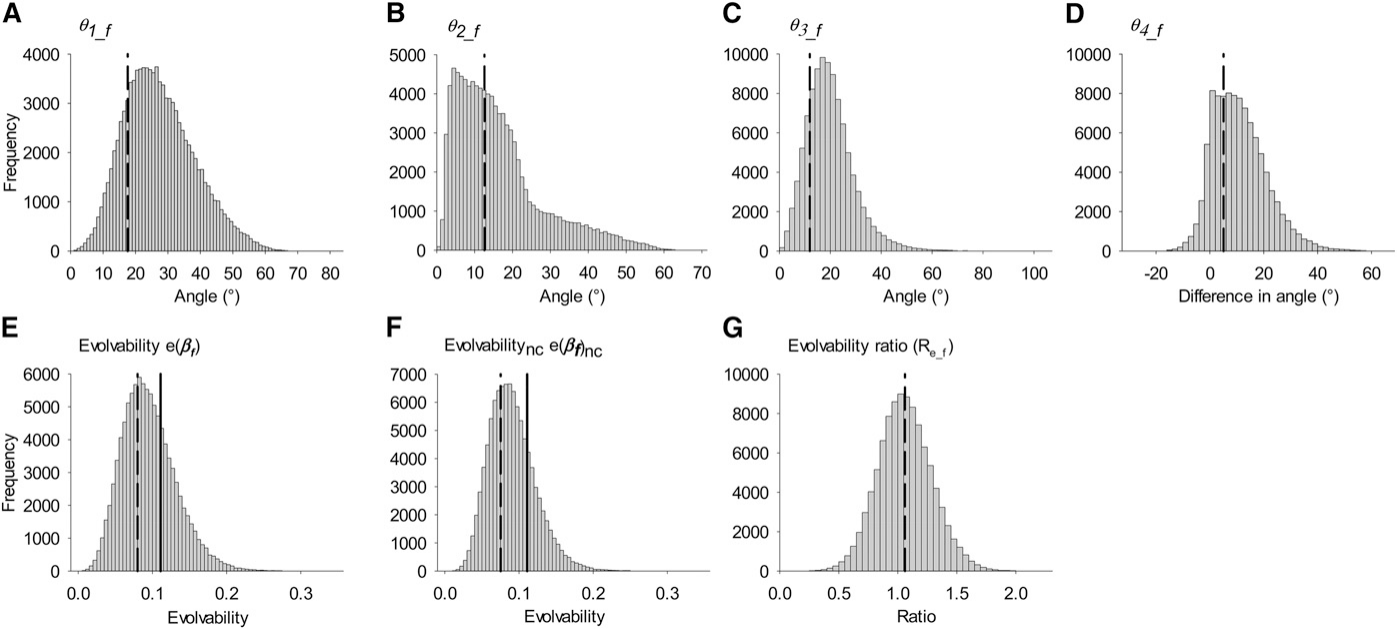
The simulated distribution of estimates of *θ* and *e*(**β**) for females. (A) *θ*_1_f_; (B) *θ*_2_f_; (C) *θ*_3_f_; (D) *θ*_4_f_; (E) *e*(**β_f_**); (F) *e*(**β_f_**)_nc_; (G) *R*_e_f_ produced by carrying through the errors in the estimation of **G_f_** and **β_f_**. Values <0 cannot exist except for *θ*_4_f_, and thus the distributions are presented to aid in interpretation of whether the simulated distributions are distinct from zero, *i.e.*, have a normal distribution that is not highly concentrated near (ramped up against) zero. Dashed lines show the position of the “best estimate”, *i.e.*, the estimate when using the maximum-likelihood estimate of the parameters of **G_f_** and **β_f_**; this is the value given in Table 2 and Table 3. For E and F, solid lines show the position of the average evolvability over random selection gradients (*ē*_f_); see *Materials and Methods* for details.

**Figure 3:**
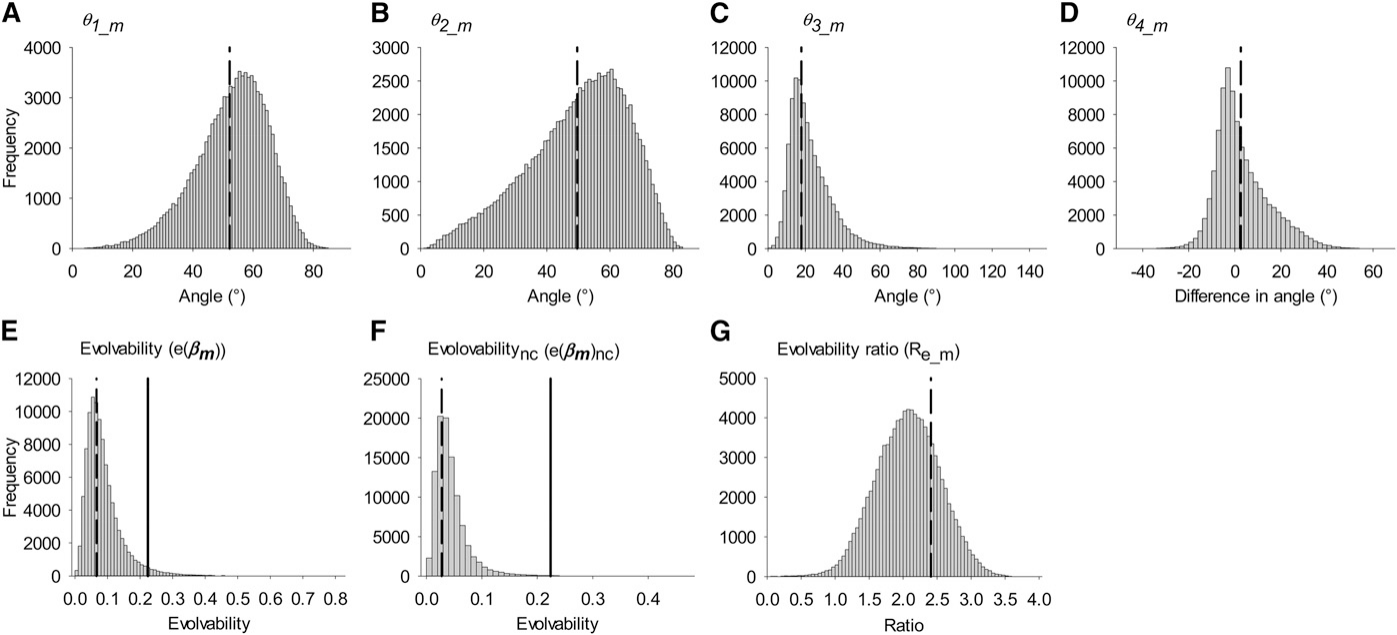
The simulated distribution of estimates of *θ* and *e*(**β**) for males. (A) *θ*_1_m_; (B) *θ*_2_m_; (C) *θ*_3_m_; (D) *θ*_4_m_; (E) *e*(**β_m_**); (F) *e*(**β_m_**)_nc_; (G) *R*_e_m_. Values <0 cannot exist except for *θ*_4_m_. Dashed lines show the position of the “best estimate,” Solid lines show the position of the average evolvability over random selection gradients (*ē*_m_); see *Materials and Methods* for details.

**Figure 4:**
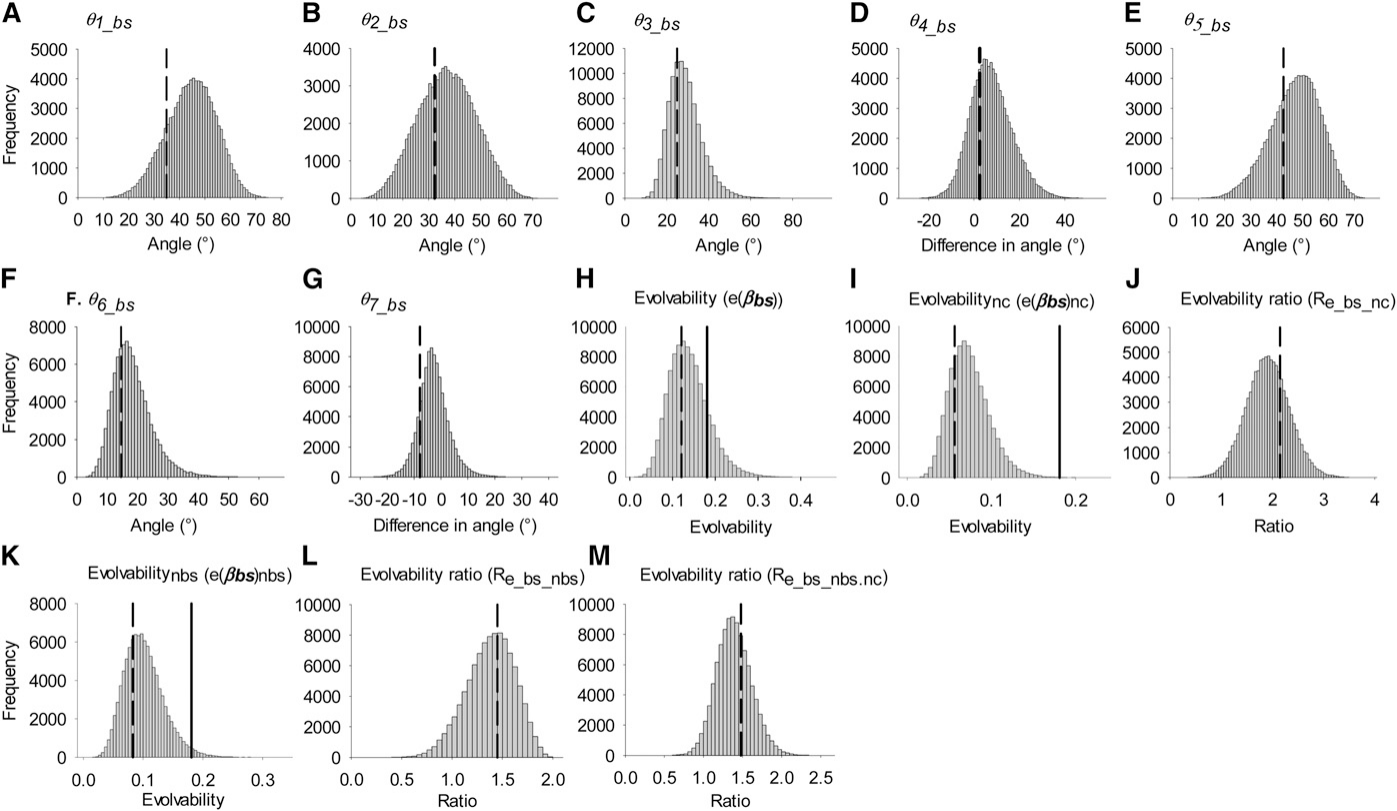
The simulated distribution of estimates of *θ* and *e*(**β**) for both-sex models. (A) *θ*_1_bs_; (B) *θ*_2_bs_; (C) *θ*_3_bs_; (D) *θ*_4_bs_; (E) *θ*_5_bs_; (F) *θ*_6_bs_; (G) *θ*_7_bs_; (H) *e*(*β*_bs_); (I) *e*(*β*_bs_)_nc_; (J) *R*_e_bs_nc_, the ratio *e*(**β_bs_**)/*e*(**β_bs_**)_nc_; (K) *e*(*β*_bs_)_nbs_; (L) *R*_e_bs_nbs_, the ratio *e*(**β_bs_**)/*e*(**β_bs_**)_nbs_; (M) *R*_e_bs_nbs.nc_, the ratio *e*(**β_bs_**)_nbs_/*e*(**β_bs_**)_nc_. Values <0 cannot exist except for *θ*_4_bs_ and *θ*_7_bs_. Dashed lines show the position of the “best estimate.” Solid lines show the position of the average evolvability over random selection gradients (*ē*_bs_); see *Materials and Methods* for details.

Estimates of **β** and **G** have associated error and thus so do values calculated from them [*e.g.*, $\Delta \bar{z}$, *θ*, and *e*(**β**)]. Errors in these estimates were approximated using an MC simulation algorithm (see also Morrissey *et al.* 2012a). Briefly, we drew 100,000 multivariate random normal (MVN) values of **S**, **P**, and **G**, using the maximum-likelihood estimates of these parameters (from ASReml) as the mean and the variance covariance matrices of these parameter estimates as the variance (again these are given in ASReml). These 100,000 values were then combined as appropriate in Equations 5, 4, 6, 7, and 9 to produce 100,000 estimates of **β**, $\Delta \bar{z}$, *θ*, *e*(**β**), and *R*_e_. The 95% credible interval (CI) around these values was then calculated using the quantile function in R and used as an estimate of the 95% credible interval around each parameter estimate. It should be noted that this method assumes the sampling errors in the estimates of variances and covariances are multivariate normal. For angles *θ*_1_, *θ*_2_, *θ*_3_, *θ*_5_bs_, and *θ*_6_bs_ (which are all defined as angles between two vectors) and for all values of evolvability, estimates cannot be negative and thus interpreting a lack of overlap of the 95% CI with zero as indicative of the value differing from zero is not valid. As such, statistical hypothesis tests have limited meaning and we therefore assessed statistical support for substantially nonzero values by examining the distribution of MC samples (see Figure 2, Figure 3, and Figure 4). In practice, this involves visual inspection of the distributions of estimates and, when the distribution is concentrated close to zero (*i.e.*, is associated with left truncation and strong right skew), drawing conclusions equivalent to those associated with failure to reject a null hypothesis.

## Results

### Part 1: Variance decomposition: Estimating G

#### Univariate analysis:

There was evidence of significant additive genetic variance for all female life history traits apart from female longevity and for male ABS but not other male life history traits (Table S1). Nongenetic random effects followed expected patterns with maternal and birth year effects being significant only for early life history traits although not all early life history traits in males (Table S1).

#### Multivariate analysis:

Multivariate models of phenotypic covariance within each sex indicated positive phenotypic correlations among all traits (Table S2), with all but one significantly greater than zero. In addition, all but one estimable nongenetic covariances among traits were positive (Table S3). Full-rank FA models of the genetic covariance matrix (**G**) converged for females, but for males and both sexes the maximal-rank models that would converge were 2 (of a maximum of 4) and 4 (of 8), respectively (Table S4). Although statistical comparison provided support only for lower-rank models for all **G** matrices (Table S4), we used the highest-rank model that converged in all subsequent analyses, for the reasons described in *Materials and Methods* and File S1. Genetic covariances between traits within and between the sexes were a mix of positive and negative values, although within-sex genetic covariances in males were all positive (Table S3, Table S5, and Table S6). A principal component analysis (PCA) of **G_bs_** revealed a major axis of genetic variation that loaded positively on all male traits and female *L*, but negatively although weakly on female SBA, AFR, and ABS (Table S7C). A similar pattern was apparent when examining the sexes separately: negative associations between female longevity and the other female traits compared to positive associations among all male traits (Table S7, A and B).

### Part 2: Assessing the potential for genetic constraint to evolution

All selection differentials and gradients were positive and strong, as life history traits must be by definition (Table 1). Given such strong positive selection, visual inspection of the estimated genetic parameters (Table S3, Table S5, Table S6, and Table S8) suggested an aspect of constraint for female traits in the general pattern of negative genetic covariance between survival and reproductive traits (Table S5 and Table S8). In males, an overall pattern of facilitation of adaptive evolution dominated as estimated genetic covariances were all positive (Table S6); however, it is unclear from the multiple imprecise estimates alone whether such an interpretation is really justified. Similarly, interpretation of the multiple modest between-sex genetic correlations (Table S3) is difficult from consideration of the estimates alone, necessitating consideration of metrics that integrate over the implications of all aspects of **G** and **β**.

**Table 1:** Selection differentials (±SE) and selection gradients (95% CI) for (standardized) male and female life history traits

Trait	Selection differential (**S**)	Selection gradient (***β***)
Female SBA	**1.25 ± 0.07**	NA
Female AFR	**0.186 ± 0.037**	**0.137 (0.108, 0.167)**
Female *L*	**0.538 ± 0.046**	**0.531 (0.505, 0.558)**
Female ABS	**0.140 ± 0.022**	**0.0776 (0.0603, 0.0936)**
Male SBA	**1.71 ± 0.13**	NA
Male AFR	**0.413 ± 0.134**	0.180 (−0.042, 0.412)
Male *L*	**0.708 ± 0.134**	**0.498 (0.324, 0.672)**
Male ABS	**0.468 ± 0.063**	**0.419 (0.306, 0.515)**

Selection differentials and associated standard errors were calculated in ASReml; selection gradients were calculated using the formula $\beta =P^{-1}S$ for either sex. Because **P** was undefined between survival to breeding age (SBA) and all other traits, only selection differentials for this trait could be estimated. **P** for age at first reproduction (AFR), longevity (*L*), and annual breeding success (ABS) in both sexes is presented in Table S2. As before, note that AFR is premultiplied by −1 such that positive values indicate selection for earlier reproduction. Values in boldface type are significantly greater than zero based on either log-likelihood ratio tests comparing models with the parameter fixed to zero *vs.* estimated (selection differentials) or whether or not the 95% credible interval overlaps zero (selection gradients).

### Metrics of constraint

#### Angle of deflection (*θ*) for females:

*θ*_1_*__f_* was small [17.6°, less than midway between 0° (no constraint) and 90° (an absolute constraint)], but appeared greater than zero (the distribution is not highly concentrated near zero, Figure 2A), suggesting that unequal genetic variances and/or nonzero genetic covariances deflected the direction of the predicted response to selection away from the direction of the vector of selection, but by a small amount. *θ*_2_*__f_*, the effect of unequal genetic variances alone, was also small (12.6°, Table 2, Figure 2B) but in this case the simulated distribution was highly concentrated near zero, suggesting little evidence that unequal genetic variances deflect $\Delta \bar{z}$ from **β**. *θ*_3_*__f_*, the effect of nonzero genetic covariances on the direction of the predicted response to selection, was 11.9° (Table 2) and distinct from zero (Figure 2C), suggesting genetic covariances have a mild effect on the predicted response to selection. Finally, *θ*_4_*__f_* (the difference between *θ*_1_*__f_* and *θ*_2_*__f_*) was merely 5.06° (Table 2, Figure 2D) and the 95% credible interval overlapped zero.

**Table 2:** Estimates of deflection (*θ*) for females, males, and both sexes

Parameter	Description	Angle (°)	95% CI
Females			
* θ*_1_f_	Angle between $\Delta \bar{z}$**_f_** and **β_f_**, effect of unequal variances and nonzero within-sex covariances	17.6	9.46–50.8
* θ*_2_f_	Angle between $\Delta \bar{z}$**_fnc_** and **β_f_**, effect of unequal variances	12.6	2.66–46.9
* θ*_3_f_	Angle between $\Delta \bar{z}$**_f_** and $\Delta \bar{z}$**_fnc_**, effect of within-sex covariances on the direction of the response to selection	11.9	5.36–41.6
* θ*_4_f_	*θ*_1_ − *θ*_2_, effect of within-sex covariances on constraint*^a^*	5.06	−4.36–33.2
Males			
* θ*_1_m_	Angle between $\Delta \bar{z}$**_m_** and **β_m_**, effect of unequal variances and nonzero within-sex covariances	52.2	26.2–72.9
* θ*_2_m_	Angle between $\Delta \bar{z}$**_mnc_** and ***β_m_***, effect of unequal variances	49.6	15.0–74.6
* θ*_3_m_	Angle between $\Delta \bar{z}$**_m_** and $\Delta \bar{z}$**_mnc_**, effect of within-sex covariances on the direction of the response to selection	17.9	8.17–53.0
* θ*_4_m_	*θ*_1_ − *θ*_2_, effect of within-sex covariances on constraint*^a^*	2.57	−13.8–31.2
Both sexes			
* θ*_1_bs_	Angle between $\Delta \bar{z}$**_bs_** and **β_bs_**, effect of unequal variances and nonzero within- and between-sex covariances	34.9	24.5–62.5
* θ*_2_bs_	Angle between $\Delta \bar{z}$**_bsnc_** and **β_bs_**, effect of unequal variances	32.3	15.9–58.1
* θ*_3_bs_	Angle between $\Delta \bar{z}$**_bs_** and $\Delta \bar{z}$**_bsnc_**, effect of within- and between-sex covariances on the direction of the response to selection	24.9	16.3–47.5
* θ*_4_bs_	*θ*_1_ − *θ*_2_, effect of nonzero within- and between-sex covariances on constraint*^a^*	2.54	−8.91–27.5
* θ*_5_bs_	Angle between $\Delta \bar{z}$**_nbs_** and **β_bs_**, effect of unequal variances and nonzero within-sex covariances	42.7	26.9–64.0
* θ*_6_bs_	Angle between $\Delta \bar{z}$**_bs_** and $\Delta \bar{z}$**_nbs_**, effect of between-sex covariances on the direction of the response to selection	14.6	8.43–33.3
* θ*_7_bs_	*θ*_1_ – *θ*_5_, effect of nonzero between-sex covariances on constraint*^a^*	−7.80	−13.0–9.73

Ninety-five percent credible intervals were calculated by simulation as described in *Materials and Methods*.

a Positive values suggest covariances increase constraint.

#### Evolvability for females:

The evolvability of female traits when considering genetic variances and covariances [*e*(**β_f_**)] was 0.0801 (Table 3, Figure 2E). The distribution of *e*(**β_f_**) (Figure 2E) suggested this value was distinct from 0, but not from the average evolvability ($\bar{e}$_f_) of 0.111. The evolvability of female traits due to genetic variances alone [*e*(**β_f_**)_nc_] was 0.0753 (Table 3) and appeared distinct from 0, but not from $\bar{e}$_f_ (Figure 2F). *R*_e_f_, the ratio of *e*(**β_f_**)/*e*(**β_f_**)_nc_, showed no evidence of differing from 1 (*R*_e_f_ = 1.06; 95% CI = 0.63–1.53, Table 3, Figure 2G), implying little effect of genetic covariances between female traits on evolvability.

**Table 3:** Measures of evolvability [*e*(β)] and the ratio of evolvability calculated with and without genetic covariances (*R*_e_) for female (_f_), male (_m_), and both-sex (_bs_) models

Description	Estimate (95% CI)
Female	
Evolvability *e*(**β_f_**)	0.0801 (0.0363, 0.177)
Evolvability_nc_ *e*(**β_f_**)_nc_	0.0753 (0.0396, 0.162)
Evolvability ratio *R*_e_f_ [*e*(**β_f_**)/*e*(**β_f_**)_nc_]	1.06 (0.631, 1.53)
Male	
Evolvability *e*(**β_m_**)	0.0659 (0.0218, 0.235)
Evolvability_nc_ *e*(**β_m_**)_nc_	0.0274 (0.0106, 0.120)
Evolvability ratio *R*_e_m_ [*e*(**β_m_**)/*e*(**β_m_**)_nc_]	2.41 (1.18, 2.97)
Both sexes	
Evolvability *e*(**β_bs_**)	0.121 (0.0626, 0.244)
Evolvability_nc_ *e*(**β_bs_**)_nc_	0.0561 (0.0350, 0.128)
Evolvability ratio *R*_e_bs_nc_ [*e*(**β_bs_**)/*e*(**β_bs_**)_nc_]	2.15 (1.11, 2.75)
Evolvability_nbs_ *e*(**β_bs_**)_nbs_	0.0829 (0.0475, 0.176)
Evolvability ratio *R*_e_bs_nbs_ [*e*(**β_bs_**)/*e*(**β_bs_**)_nbs_]	1.45 (0.883, 1.79)
Evolvability ratio *R*_e_bs_nbs.nc_ [*e*(**β_bs_**)_nbs_/*e*(**β_bs_**)_nc_]	1.48 (0.981, 1.86)

Evolvability and the 95% CIs are calculated as described in Materials and Methods. _nc_, all genetic correlations fixed to 0; _nbs_, between-sex genetic correlations fixed to 0.

#### Angle of deflection (*θ*) for males:

*θ*_1__m was intermediate in magnitude (52.2°, Table 2, Figure 3A) and its distribution was distinct from zero (Figure 3A). The effect of unequal genetic variances alone was similar in magnitude (*θ*_2__m = 49.6°, Table 2) and again appeared distinct from 0 (Figure 3B), suggesting significant deflection. The effect of genetic covariances on the direction of $\Delta \bar{z}$**_m_** (*θ*_3_m_) was small (17.9°, Table 2), but the simulated distribution (Figure 3C) suggested this value was distinct from zero and thus that genetic covariances altered the direction of the predicted response to selection in males. There was no evidence of genetic covariances increasing constraint: *θ*_4_m_ (the difference between *θ*_1_m_ and *θ*_2_m_) was only 2.57° (95% CI = −13.8–31.2, Table 2, Figure 3D).

#### Evolvability for males:

The evolvability of male traits when considering genetic variances and covariances [*e*(**β_m_**)] was 0.0659 (Table 3) and appeared distinct from 0 but not quite from $\bar{e}$_m_ [Figure 3E, $\bar{e}$_m_ = 0.224, upper 95% CI of *e*(**β_m_**) = 0.235]. The evolvability of male traits when considering only genetic variances [*e*(**β**_m_)_nc_] was 0.0274 (Table 3) and appeared distinct from 0 and also <$\bar{e}$_m_ (Figure 3F). *R*_e_m_, the ratio *e*(**β_m_**)/*e*(**β_m_**)_nc_, was significantly >1 (2.41; 95% CI = 1.18–2.97, Table 3, Figure 3G,), suggesting that genetic covariances between male life history traits significantly increased evolvability and thus facilitated the predicted evolutionary response.

#### Angle of deflection (*θ*) combining both sexes:

Angles for the both-sex analysis are presented in Table 2 and Figure 4, A–G. The general pattern was one of the vector of the predicted response to selection being deflected away from the vector of selection by a moderate amount [*e.g.*, *θ*_1_*___*_bs_ = 34.9° (Table 2), and *θ*_1_bs_ appeared distinct from zero (Figure 4A)], but that within- and between-sex genetic covariances did not increase deflection and thus constraint (*θ*_4_bs_ = 2.54°; 95% CI = −8.91–27.5, Table 2, Figure 4D). Unequal genetic variances alone caused deflection of intermediate magnitude (*θ*_2_bs_ = 32.3°, Table 2, Figure 4B). Although between-sex covariances appeared to alter the direction of the predicted response to selection (*θ*_6_bs_ = 14.6°, Table 2, Figure 4F), there was little evidence that between-sex covariances increased constraint in terms of *θ* (*θ*_7_bs_ = −7.80°, Table 2, Figure 4G).

#### Evolvability combining both sexes:

Predictions of evolvability are presented in Table 3 and Figure 4, H–M. In general, evolvability was >0, but lower than the average evolvability over random selection gradients ($\bar{e}$_bs_) (Figure 4, H, I, and K). However, there was no evidence that genetic covariances caused a reduction in evolvability (*i.e.*, increase in constraint). Instead, evolvability was higher when including genetic covariances than when fixing them to zero (Figure 4, J, L, and M; *R*_e_bs_nc_ = 2.15, 95% CI = 1.11–2.75; *R*_e_bs_nbs_ = 1.45, 95% CI = 0.883–1.79; *R*_e_bs_nbs.nc_ = 1.48, 95% CI = 0.981–1.86, although the 95% CIs of both *R*_e_bs_nbs_ and *R*_e_bs_nbs.nc_ overlapped 1; see Table 3). Thus the positive effects of covariances on evolvability appeared to be due to both between- and within-sex covariances.

## Discussion

These results provide a detailed investigation of genetic constraints to life history evolution in a wild population of red deer. Studies that apply multiple measures of genetic constraint, particularly measures of evolvability, to data from a wild animal population are extremely rare. Teplitsky *et al.* (2014) assess multivariate evolvability, and also the effect of genetic correlations on the predicted rate of adaptation, in an analysis of morphological traits in 10 different bird populations and found general nonalignment between selection and genetic variance (see also Simonsen and Stinchcombe 2011 for a field study in plants and Morrissey *et al.* 2012b, but note Morrissey *et al.* do not present explicit estimates of evolvability). In contrast, in our analyses, although a substantial proportion of the estimates of individual genetic covariances were negative (11/28; Table S3), in general we found overall genetic constraints to be relatively mild and to result mainly from genetic variances rather than from genetic covariances among traits. In particular, we found little evidence that genetic covariances among traits in males or between the sexes generate constraint; rather, genetic covariances in males and between the sexes appear to facilitate the predicted response to selection in terms of evolvability and any constraint occurs primarily from the pattern of genetic variances.

### Measures of constraint

In general, the deflection of the predicted response to selection away from the vector of selection (*i.e.*, the direction of fastest adaptation) was small. This was particularly true in females (*θ*_1_f_ and *θ*_2_f_ < 20°), while in the male and both-sex models, deflection was of intermediate magnitude (*θ*_1_m_, *θ*_1_bs_, *θ*_2_m_, and *θ*_2_bs_ >30° but <53°). In females the small deflection and thus constraint that was evident appeared to result from a combination of unequal genetic variances and nonzero genetic covariances, since neither one caused significant deflection alone. However, in males and both-sex models, genetic covariances caused limited deflection, while unequal genetic variances caused significant (albeit still not large) deflection and thus constraint.

Conclusions regarding the extent of constraint were slightly different when considering evolvability. For female and both-sex models, evolvability was similar to the average evolvability, a baseline that indicates the evolutionary potential of **G** independent of the direction of selection relative to **G** (Hansen and Houle 2008; Innocenti and Chenoweth 2013). Thus, the pattern of genetic variances and covariances relative to the direction of selection does not appear to greatly restrict the evolutionary potential of females or both sexes combined in comparison to the evolutionary potential of **G** [*e*(**β_f_**) was 72% of $\bar{e}$_f_, *e*(**β_bs_**)_nc_ was 67% of $\bar{e}$_bs_]. However, for males, evolvability was low compared to the average evolvability particularly when genetic covariances were fixed to zero [*e*(**β_m_**) was 29% of $\bar{e}$_m_ and *e*(**β_m_**)_nc_ was 12% of $\bar{e}$_m_]. This suggests constraint in the pattern of genetic variances relative to the direction of selection in males when compared to the evolutionary potential of **G**. The effect of genetic covariances on the predicted evolvability differed slightly between female *vs.* male and both-sex models. For females, genetic covariances had very little effect on the predicted evolvability, as opposed to the slight increase in deflection and thus constraint as measured by deflection. In male and both-sex models evolvability increased as a result of genetic covariances and thus, while causing only a very slight increase in evolvability in absolute terms [*i.e.*, small changes in *e*(**β**)], caused a large increase in evolvability in relative terms (*R*_e_m_ and *R*_e_bs_nc_ estimates were >2). This suggests that genetic covariances increased the predicted evolvability when considering males and both sexes, by increasing the magnitude of the predicted response in the direction of maximally increasing fitness.

### Comparison with other results from the Rum red deer population

Detailed discussion of the comparison with previous results from the Rum red deer population is given in File S1. However, it should be noted here that despite patterns of genetic covariances between female traits being similar to those of a previous study (Morrissey *et al.* 2012b), the results presented in the present study provide weaker evidence for genetic constraint than that in two previous studies (Foerster *et al.* 2007; Morrissey *et al.* 2012b). These differences appear to be driven by the treatment of survival to breeding age, pointing to parent–offspring patterns/processes being a potential key area for future study of genetic constraints in this population (see File S1 for further discussion).

### Comparison with results from other populations

Our results provide evidence for relatively modest genetic constraint to evolution, particularly as a result of genetic covariances between traits. Although rare, other estimates of multivariate genetic constraint in the literature have provided stronger evidence of constraint (Blows *et al.* 2004; Hine and Blows 2006; Smith and Rausher 2008; Lewis *et al.* 2011; Simonsen and Stinchcombe 2011; Gosden *et al.* 2012; Williams *et al.* 2012; Teplitsky *et al.* 2014). The reason for the difference in the magnitude of constraint between our results and those of previous studies is difficult to assess, given the multivariate nature of the techniques. Previous studies have tended to focus on combinations of traits, among which one might predict strong correlations. For example, a number of studies focus on *Drosophila* cuticular hydrocarbons (Blows *et al.* 2004; Gosden *et al.* 2012), many of which are built from the same amino acids and thus might be expected to share biosynthetic pathways (Blows *et al.* 2004); in their large-scale analyses of 10 bird populations, Teplitsky *et al.* (2014) considered four different morphological traits, all of which were positively correlated. Our study focuses on different components of fitness that might not be expected to be functionally related—at least not via immediate and simple biochemical relationships. Having said this, Lewis *et al.* (2011) analyze life history traits including development time and longevity in the Indian meal moth and also find evidence of strong constraint.

In addition, our results are from a wild rather than a laboratory population. While very useful, laboratory studies necessarily deal with populations that are experiencing selection pressures that are not “natural” selection and that they may have experienced for only a limited time period compared to wild populations; alternatively, selection pressures may have been very consistent compared to those experienced in wild populations. Thus differences in the patterns of selection between laboratory and natural populations may also contribute to differences in results. For example, a recent study of diet preferences in a population of *Drosophila melanogaster* that was not laboratory adapted found weak evidence for multivariate constraint (Reddiex *et al.* 2013). Teplitsky *et al.*’s (2014) analysis suggests multivariate constraints to the evolution of morphological traits in wild avian populations, but this appears to be due to antagonistic selection on positively genetically correlated traits. In one other study of a wild population, Coltman *et al.* (2005) analyze covariation in life history traits in bighorn sheep (*Ovis canadensis*) and find substantial angles between **β** and the first three principal components of **G**: 117°, 73°, and 103°. Applying the same technique to the results in our study (PC1–3 in Table S7C compared to the vector of selection gradients in Table 1) gives angles of 76°, 68°, and 121°, which would perhaps suggest a stronger genetic constraint. However, when considering the effect of **G** on deflection and evolvability, genetic constraint is less apparent. Our study therefore illustrates the difference in conclusions that may be drawn from alternative approaches to quantifying evolutionary constraints and argues for a range of different metrics to be explored (see, for example, Simonsen and Stinchcombe 2011). Given the overall paucity of data on this subject, and the potential tendency for significant rather than nonsignificant evidence of constraint to be published earlier, it will be interesting to see the patterns that emerge from future studies in wild populations.

The results of our study suggest a lack of genetic constraint to the evolution of life history traits in this population and hence to the maintenance of genetic variance in the direction of selection. As such, traits should have the potential to respond rapidly to natural selection and yet a lack of response to natural selection is commonly observed in wild populations (*e.g.*, Kruuk *et al.* 2001; Merilä *et al.* 2001). Explaining the maintenance of genetic variance for quantitative traits is a core area of research in evolutionary biology and a number of potential explanations, other than multivariate genetic constraint, have been proposed (Via and Lande 1985; Gillespie and Turelli 1989; Charlesworth and Hughes 2000; Johnson and Barton 2005). Of particular relevance in natural populations may be the presence of environmental variation and thus the potential for genotype-by-environment interactions and variable selection to maintain genetic variation (Gillespie and Turelli 1989; Charmantier and Garant 2005; Bell 2010; Morrissey and Hadfield 2012). Certainly there is the potential for environmental variation both temporally and spatially within the red deer population studied here (Coulson *et al.* 1997; Stopher *et al.* 2012) and thus for genotype-by-environment interactions and variable selection to be important in the maintenance of genetic variation for quantitative traits. Given the current lack of data on microevolutionary parameters in natural populations and how they vary with environment (but see, for example, Charmantier and Garant 2005; Wilson *et al.* 2006; Robinson *et al.* 2009), further research into this area would be of interest. However, it should be noted that current evidence suggests patterns of directional selection are remarkably stable across study systems, although this pattern does not necessarily hold within any particular population (Siepielski *et al.* 2009; Morrissey and Hadfield 2012).

Finally, it is worth clarifying here that multiple regression-based selection analysis is expected to provide robust inference when complete life history data are analyzed. As we and others have discussed in the past, multiple regression-based selection gradient estimates may fail to represent the true direct effects of traits on fitness when unmeasured variables cause trait–fitness covariance (Robertson 1966; Rausher 1992; Kruuk *et al.* 2003; Morrissey *et al.* 2010, 2012a). Two alternative strategies are available to ensure that quantitative genetic predictions of evolutionary trajectories are robust. First, one might seek to estimate the additive genetic covariances of traits with relative fitness, which gives complete prediction of evolutionary change (Robertson 1966; reviewed in Morrissey *et al.* 2010). However, estimation of the genetic covariance of traits with relative fitness, despite being an application of the “secondary theorem of selection” (Robertson 1968), actually tells us very little about the phenotypic process of selection—a trait that covaries genetically with relative fitness may not itself be selected at all (for example, the trait may not *causally* influence fitness but may be genetically correlated with a trait that does; discussed in Morrissey *et al.* 2010, 2012a; Morrissey 2014). The second strategy is to include sufficient variables in multiple-regression analyses to account for all trait–fitness covariance. These variables may, for example, be features of the environment that ultimately cause trait–fitness covariance. Alternatively, the included traits may not necessarily be those that ultimately *cause* trait–fitness variation, but rather phenotypic traits by which the effects of the causal traits are mediated. For example, if an environmental variable *E* causes variation in survival *S* and reproduction *R*, regression of relative fitness *w* on *S* will give the wrong selection gradient for *S*. However, analyses regressing either *w* on *S* and *E* or *w* on *S* and *R* (even without the ultimate source of covariance, *E*) will give correct selection gradient estimates for *S* and in the latter case, the bonus of the correct selection gradient for R; in each case correct refers to the value of the selection gradient that provides the correct evolutionary predictions when used in the Lande equation (this may be initially unintuitive; for a simple demonstration, see R console procedure at end of paragraph). Thus, while it is typically impossible to be sure that all relevant traits are included, a comprehensive set of life history traits (*e.g.*, survival, age at first reproduction, annual reproduction, in both sexes, as herein) is a special case where all pathways mediating any effects of traits or environmental variables on fitness are necessarily included, because life history completely determines fitness; there are no missing traits, in the sense that all effects of variables on fitness are mediated by life history.

Enter the following in an R console:

#simulate data according to hypothetical example in text, true LH betas = 1

n<-10000; E<-rnorm(n,0,1); S<-1*E+rnorm(n,0,1); R<-1*E+rnorm(n,0,1); w<-1*R+1*S+rnorm(n,1,1);

#missing variable problem

summary(lm(w∼S))

#solution 1: include ultimate effects

summary(lm(w∼S+E))

#solution 2: include mediating effects (complete life history)

summary(lm(w∼S+R)).

### Conclusions

This study attempts to quantify the multivariate genetic constraint, considering both within- and between-sex genetic (co)variation, in a wild population experiencing a natural environment. Overall we found estimates of genetic constraint were mild and that patterns of genetic variances rather than genetic covariances were the main source of genetic constraint. There was little support for the contention that between-sex genetic covariances caused constraint, indicating that sexual antagonism may not be as strong as previous results from this population suggested. Finally, the degree of genetic constraint in this study was much lower than in previous studies based predominantly on laboratory populations. Given the lack of data on natural populations and the difficulty of estimating these parameters in such populations, we encourage further analysis of this type to assess whether the apparently lower level of genetic constraint in natural populations is a general pattern.

## Supplementary Material

Supporting Information
