# Supplementary material for: A Multivariate Analysis of Genetic Constraints to Life History Evolution in a Wild Population of Red Deer
Source: Genetics. 2014 Oct 2;198(4):1735–49. doi: 10.1534/genetics.114.164319 (PMC4256783; doi:10.1534/genetics.114.164319)
Supplement: Supporting Information [file supp_198_4_1735__index.html]

A Multivariate Analysis of Genetic Constraints to Life History Evolution in a Wild Population of Red Deer — Supporting Information 

# A Multivariate Analysis of Genetic Constraints to Life History Evolution in a Wild Population of Red Deer

**Files in this data supplement:**

- Supporting Information: File S1 and Tables S1-S8 (PDF, 237 KB)
- File S1: Methods and Discussion (PDF, 186 KB)
- Table S1: Estimates of variance components for female and male life history traits from univariate models. (PDF, 150 KB)
- Table S2: Phenotypic variance-covariance matrix for (standardized) male and female life history traits. (PDF, 138 KB)
- Table S3: Model showing variances (diagonal), covariances (below diagonal) and correlations (above diagonal) (plus or minus 1SE) for the minimal models of life history traits in both sexes. (PDF, 154 KB)
- Table S4: FA models of a) female, b) male and c) both-sex G-matrices. (PDF, 176 KB)
- Table S5: Female (co)variance components. (PDF, 152 KB)
- Table S6: Male (co)variance components. (PDF, 140 KB)
- Table S7: Principal components analysis (PCA) of **G**-hat estimated from the maximal FA model possible for a) female and b) male and c) both-sex genetic variance-covariance matrices. (PDF, 152 KB)
- Table S8: Female genetic (co)variance components from a non-factor analytic multivariate model of all female traits simultaneously. (PDF, 138 KB)
