# Supplementary material for: A Multivariate Analysis of Genetic Constraints to Life History Evolution in a Wild Population of Red Deer
Source: Genetics. 2014 Oct 2;198(4):1735–49. doi: 10.1534/genetics.114.164319 (PMC4256783; doi:10.1534/genetics.114.164319)
Supplement: Supporting Information [file f077415ec3a822cc8d3d5281a1fc870c_genetics.114.164319-1.pdf]

File S1

Methods and Discussion

Fixed effects: Fixed effects known to be important in this system were included in models of each of the different life history traits, as follows:

For Survival to Breeding Age (SBA), we included linear and quadratic effects of the mother's age (Coulson *et al.* 2003), mother's population sub-area in the offspring's first two years of life (to account for variation in habitat quality between four different sub-areas of the study site (Coulson *et al.* 1997)) and mother's recent reproductive history (whether or not the female had given birth to a calf the previous year and whether it had survived its first year, five different levels; Naive (N), female had not bred previously; True yeld (TY), female had bred previously but did not breed in the previous year; Summer yeld (SY), female bred in the previous year but the calf died before 1 October; Winter yeld (WY), female bred in the previous year but the calf died between 1 October and 1 May; Milk (M), the female successfully reared a calf in the previous year, for details see (Clutton-Brock *et al.* 1983)).

For Age at First Reproduction (AFR), in females we included an individual's mother's population sub-area in her first two years of life (to account for early life differences in habitat quality, four levels as for SBA). For males this fixed effect was not significant and was thus removed.

For Longevity (L), we included a female's lifetime population sub-area as the area in which she spent most years of her life, whereas for males such information was not available for a large number of individuals and so no fixed effects were included.

Finally, for Annual Breeding Succcess (ABS), for females, we included the fixed effects of a female's age, its quadratic, and recent reproductive history as defined for SBA. For male ABS, age and its quadratic were fitted as fixed effects.

Factor Analytic modelling: Estimating a multivariate **G**-matrix can be difficult because of the number of parameters to be estimated (Kirkpatrick and Meyer 2004; Meyer and Kirkpatrick 2005), a problem which may be exacerbated when using the incomplete pedigrees and modest sample sizes typical of data from natural populations. In an attempt to overcome these issues, we used factor analytic modeling techniques (FA) (Wright 1932; Thompson *et al.* 2003; Kirkpatrick and Meyer 2004; Meyer and Kirkpatrick 2005) to provide a (reduced rank) multivariate estimate of genetic variance-covariance matrixes, considering first either sex separately and then all eight traits across both sexes jointly. FA allows the estimation of the major independent axes of genetic variance in the traits, with each successive axis explaining decreasing variance in **G** allowing a "building-up" approach to modeling **G**: increasing numbers of genetic factors are fitted until either the fitting of additional factors is no longer possible or the model is "full rank" and contains as many genetic factors as traits (see below). By taking a FA approach we can estimate the maximal amount of variation in **G** possible given the constraints of the data.

FA involves modeling the genetic variance-covariance matrix (**G**) as a product of a number *m* of independent linear combinations of the original (*p*) traits such that:

$$\hat{\mathbf{G}} = \mathbf{\Lambda}\mathbf{\Lambda}^{\mathrm{T}} + \boldsymbol{\Psi} \qquad (2)$$

where $\hat{\mathbf{G}}$ = a (potentially reduced-rank) estimate of **G**, $\mathbf{\Lambda}$ is a lower triangle matrix of constants that represent loadings of each trait on each factor, ^{T} is the transpose of a matrix and $\boldsymbol{\Psi}$ is a vector of specific variances (Meyer and Kirkpatrick 2008). Factor analysis

becomes similar to a principal components analysis (PCA) when $\boldsymbol{\Psi}$ are fixed to zero such that:

$$\hat{\mathbf{G}} = \mathbf{\Lambda}\mathbf{\Lambda}^{\mathbf{T}} \tag{3}$$

Both forms of FA can be performed in ASReml (Thompson *et al.* 2003; Gilmour *et al.* 2009) and the significance of additional factors can be assessed by comparing the log-likelihoods of models with sequentially more (or fewer) factors. The number of degrees of freedom for each model is given by $m(2p-m+1)/2$ in which p and m are the number of traits and factors respectively. Significance is assessed from twice the difference between the log-likelihoods of successive models, assumed to be chi-squared distributed with degrees of freedom (df) equal to the change in df between models. A full rank FA model, with $\mathbf{\Lambda}$ representing a lower triangle of a matrix of dimension p (for equation (3)), is equivalent to a standard multivariate model of **G**.

Although the majority of previous approaches using FA have focused on assessing the rank of **G** (e.g. Mezey and Houle 2005; Hine and Blows 2006; Mcguigan and Blows 2007; Schroderus *et al.* 2010), it has been demonstrated that sampling variance results in an underestimate of the contribution of the smallest and an overestimate of the contribution of the largest "factor" (or eigenvector), and thus an underestimate of the rank of **G** (Hill and Thompson 1978; Meyer and Kirkpatrick 2008); which is particularly apparent for traits with lower heritability (Hine and Blows 2006). We note also that the number of factors with statistical support will depend on the statistical power of the dataset, and thus that a smaller sample size is likely to result in a conclusion that **G** is of lower rank than with a larger sample size. To avoid these issues we took an alternative approach of "building-up" an FA model, adding additional factors until either **G** was full rank (rank $\mathbf{\Lambda}$ = p (four (within-sex models) or eight (both-sex models) in this case)) or models including

additional factors were not possible (due to failure of convergence). FA allows estimation of $\hat{\mathbf{G}}$ (i.e. $\mathbf{\Lambda\Lambda}^{\mathrm{T}}$) that contains the maximum possible variance estimable given the data and thus the best possible estimate of **G** to subsequently assess its potential to generate evolutionary constraint (see below). Because the leading factors to be estimated are those that contain the most variance, any unestimable factors in our analysis should explain considerably less variance than those that are estimable and should thus have a much smaller effect on the response to selection than those that are included.

Standard genetic parameter estimates (variances and covariances of the traits) derived from FA models (using equation 3) do not have associated standard errors as the errors estimated are associated with the elements of the factors (i.e. elements of $\mathbf{\Lambda}$) rather than the elements of the recovered $\hat{\mathbf{G}}$. A principal components analysis (PCA) of $\hat{\mathbf{G}}$ (effectively **G** if analyses are full rank) allows presentation of the results of FA models in the more familiar format of eigenvalues and eigenvectors (Schroderus *et al.* 2010).

To assess the informativeness of FA models, where possible we estimated the proportion of total genetic variation explained by different models. Assessing the proportion of genetic variation explained requires deciding on a "best estimate" of the total variance in the traits. Where full rank FA models can be estimated, this was simply the trace of the estimated **G** (i.e. the sum of the genetic variances). Where full rank FA models were not possible, we used the sum of the univariate estimates of the genetic variances. Thus for females the trace of the full rank estimate of $\mathbf{G_f}$ was used, whereas for males, where a full rank model of $\mathbf{G_m}$ would not converge (see below), the sum of the univariate estimates of the genetic variances was used. For $\mathbf{G_{bs}}$ we summed our "best estimates" of the variance in $\mathbf{G_f}$ and $\mathbf{G_m}$. When covariance exists between traits, information about the variance in one

trait can be used to inform estimates of variance in other traits. As such, multivariate models may provide better estimates of the variance in a trait than univariate models and thus it is possible for even reduced rank FA models to explain more variance in **G**, and equally for full rank FA models to explain less variance in **G**, than the sum of the variances obtained from univariate models.

DISCUSSION

Comparison with other results from the Rum red deer population

Three other studies have considered the role of genetic covariances between traits and the prevalence of evolutionary constraints in the Rum red deer study population (Foerster *et al.* 2007; Morrissey *et al.* 2012; Kruuk *et al.* 2014). The overall pattern of negative genetic covariances between female survival and reproductive traits is very similar to that of a previous study on the same population (Morrissey *et al.* 2012). However, there is a difference in the evolvability ratios of female traits between these two studies (R_e = 0.63 in (Morrissey *et al.* 2012) versus 1.06 here). Furthermore, the current study provides little evidence for genetic constraint acting through between sex genetic covariances, whilst a previous study (Foerster *et al.* 2007) reported a strong negative genetic correlation between an estimate of male and female fitness. One major difference between these two previous studies and the current study is in the treatment of early life survival. Here, early life survival is modelled as a trait of the individual and describes survival to three years of age, whereas both previous studies (Foerster *et al.* 2007; Morrissey *et al.* 2012) modelled early life survival only to one year of age and considered it as a trait of the mother. If this trait is removed from the current study, female R_e values are remarkably similar to those of (Morrissey *et al.* 2012) (R_{e_f} = 0.68 in this study (data not shown) vs. 0.63 in (Morrissey *et al.* 2012)) – an observation that illustrates the changes in conclusions that

may arise dependent on exactly which traits are included in an analysis, and exactly how those traits are defined. Ideally, early life survival would be modelled as a trait of the individual with maternal and maternal genetic effects included to allow the estimation of maternal and direct genetic effects and their genetic covariance. However, in the current multivariate analysis this was not possible due to the complexity of the models that would be required. The differences between these studies points to parent-offspring patterns/processes being a potential key area for future study of genetic constraints in this population.

Finally, a multivariate study of sexual selection in relation to antler trait morphology in this population (Kruuk *et al.* 2014) found evidence of genetic variance underlying antler traits and also (as here) male annual breeding success, but – in a test of the potential for antler traits to respond to selection (Morrissey *et al.* 2010) – no evidence of genetic covariances between antler size or shape and the fitness measure. There was also a moderate discrepancy between the direction of maximum genetic variance (gmax) and that of the selection gradients, $\boldsymbol{\beta}$, with a posterior mode of the angle between the two vectors of 37.62° (95%CI 6.43, 62.34). Thus in relation to male fecundity selection for antler morphology, evolutionary constraints appear to be shaped by patterns of genetic covariances, rather than by the genetic variance of individual traits, but a similar pattern emerges of moderate rather than strong constraints.

REFERENCES

Clutton-Brock, T. H., F. E. Guinness and S. D. Albon, 1983 The costs of reproduction to red deer hinds. Journal of Animal Ecology **52:** 367-383.

Coulson, T., S. Albon, F. Guinness, J. Pemberton and T. Cluttonbrock, 1997 Population substructure, local density, and calf winter survival in red deer (*cervus elaphus*). Ecology **78:** 852-863.

Coulson, T., L. E. B. Kruuk, G. Tavecchia, J. M. Pemberton and T. H. Clutton-Brock, 2003 Estimating selection on neonatal traits in red deer using elasticity path analysis. Evolution **57:** 2879-2892.

Foerster, K., T. Coulson, B. C. Sheldon, J. M. Pemberton, T. H. Clutton-Brock *et al.*, 2007 Sexually antagonistic genetic variation for fitness in red deer. Nature **447:** 1107-1109.

Gilmour, A. R., B. J. Gogel, B. R. Cullis and R. Thompson, 2009 *Asreml user guide release 3.0*. VSN International Ltd, Hemel Hempstead, HP1 1ES, UK, www.vsni.co.uk.

Hill, W. G., and R. Thompson, 1978 Probabilities of non-positive definite between-group or genetic covariance matrices. Biometrics **34:** 429-439.

Hine, E., and M. W. Blows, 2006 Determining the effective dimensionality of the genetic variance-covariance matrix. Genetics **173:** 1135-1144.

Kirkpatrick, M., and K. Meyer, 2004 Direct estimation of genetic principal components: Simplified analysis of complex phenotypes. Genetics **168:** 2295-2306.

Kruuk, L. E. B., T. H. Clutton-Brock and J. M. Pemberton, 2014 Case study: Quantitative genetics and sexual selection of weaponry in a wild ungulate. in *Quantitative genetics of wild populations*. Oxford University Press, Oxford,.

Mcguigan, K., and M. W. Blows, 2007 The phenotypic and genetic covariance structure of drosophlid wings. Evolution **61:** 902-911.

Meyer, K., and M. Kirkpatrick, 2005 Restricted maximum likelihood estimation of genetic principal components and smoothed covariance matrices. Genetics Selection Evolution **37:** 1-30.

Meyer, K., and M. Kirkpatrick, 2008 Perils of parsimony: Properties of reduced-rank estimates of genetic covariance matrices. Genetics **180:** 1153-1166.

Mezey, J. G., and D. Houle, 2005 The dimensionality of genetic variation for wing shape in drosophila melanogaster. Evolution **59:** 1027-1038.

Morrissey, M. B., L. E. B. Kruuk and A. J. Wilson, 2010 The danger of applying the breeder's equation in observational studies of natural populations. Journal of Evolutionary Biology **23:** 2277-2288.

Morrissey, M. B., C. A. Walling, A. J. Wilson, J. M. Pemberton, T. H. Clutton-Brock *et al.*, 2012 Genetic analysis of life-history constraint and evolution in a wild ungulate population. The American Naturalist **179:** E97-E114.

Schroderus, E., I. Jokinen, M. Koivula, E. Koskela, T. Mappes *et al.*, 2010 Intra- and intersexual trade-offs between testosterone and immune system: Implications for sexual and sexually antagonistic selection. The American Naturalist **176:** E90-E97.

Thompson, R., B. Cullis, A. Smith and A. Gilmour, 2003 A sparse implementation of the average information algorithm for factor analytic and reduced rank variance models. Australian & New Zealand Journal of Statistics **45:** 445-459.

Wright, S., 1932 General, group and special size factors. Genetics **17:** 603-619.

Table S1 Estimates of variance components for female and male life history traits from univariate models. N = sample sizes (Obs = number of observations, indiv = number of individuals), SBA = survival to breeding age, AFR = age at first reproduction, ABS = annual breeding success and L = adult longevity. V_A = additive genetic variation, V_M = maternal variation, V_{PE} = permanent environment variation, V_{BY} = birth year variation, V_{YR} = year of measurement variation and V_R = residual variation. min - results from models with non-significant random effects removed. m^2 and pe^2 are the proportion of phenotypic variance explained by maternal and permanent environment effects respectively. All analyses are based on standard deviation standardised data (i.e. have a variance of 1), but models include fixed effects and so V_A is not identical to heritability (h^2). Heritabilities are presented as narrow sense heritabilities, the ratio of the additive genetic variance (V_A) to phenotypic variance (V_P). Coefficients of variance are presented for all components (except year components) as $CV_X = 100 \times \frac{\sqrt{V_X}}{\overline{X}}$, where x = trait of interest and $\overline{X}$ is the mean.

	N (Obs, indiv)	mean*	SD*	V_A±SE	V_M±SE	V_{PE}±SE	V_{BY}±SE	V_{YR}±SE	V_R±SE	h^2±SE	m^2 or pe^2 ±SE	CV_A	CV_M/ CV_{PE}	CV_R
**FEM**														
SBA	1126	1.07	1	**0.16±0.06**	**0.069±0.033**	NA	**0.064±0.023**	*NA*	**0.67±0.06**	**0.17±0.06**	**0.072±0.035**	37.1	24.6	76.2
AFR	519	11.2	1	**0.17±0.09**	**0.14±0.06**	NA	**0.069±0.033**	*NA*	**0.57±0.09**	**0.18±0.09**	**0.15±0.06**	3.72	3.38	5.05
L	338	2.51	1	0.15±0.12	*NA*	*NA*	0.036±0.031	*NA*	**0.77±0.12**	0.16±0.12	*NA*	15.3	*NA*	34.9
min L		2.51	1	0.099±0.11	*NA*	*NA*	*NA*	*NA*	**0.84±0.12**	0.11±0.12	*NA*	12.6	*NA*	36.6
ABS	3859, 439	1.27	1	**0.044±0.016**	*NA*	**0.028±0.014**	0^B	**0.033±0.01**	**0.73±0.02**	**0.053±0.019**	**0.033±0.017**	16.6	13.1	67.0
min ABS		1.27	1	**0.044±0.016**	*NA*	**0.029±0.015**	*NA*	**0.033±0.010**	**0.73±0.02**	**0.053±0.018**	**0.035±0.017**	16.6	13.3	67.0
**MALES**														
SBA	1114	0.85	1	0.053±0.046	**0.060±0.030**	*NA*	**0.080±0.027**	*NA*	**0.72±0.05**	0.059±0.051	**0.066±0.032**	27.2	28.7	99.6
AFR	149	10.8	1	0.40±0.27	0.11±0.15	*NA*	0.054±0.062	*NA*	**0.46±0.24**	0.39±0.25	0.11±0.15	5.84	3.06	6.27
min AFR		10.8	1	0.48±0.27	*NA*	*NA*	*NA*	*NA*	**0.55±0.23**	0.46±0.24	*NA*	6.39	*NA*	6.85
L	245	3.69	1	0.086±0.153	*NA*	*NA*	0.049±0.042	*NA*	**0.86±0.17**	0.086±0.15	*NA*	7.93	*NA*	25.2
min L		3.69	1	0.17±0.17	*NA*	*NA*	*NA*	*NA*	**0.83±0.18**	0.17±0.17	*NA*	11.2	*NA*	24.7
ABS	2004, 570	0.58	1	**0.070±0.032**	*NA*	**0.12±0.03**	0.0085±0.0085	0.0045±0.0042	**0.65±0.023**	**0.082±0.038**	**0.14±0.04**	45.7	59.5	139
min ABS		0.58	1	**0.079±0.033**	*NA*	**0.12±0.03**	*NA*	*NA*	**0.65±0.023**	**0.093±0.038**	**0.14±0.04**	48.6	59.2	140

* NB all phenotypic data were standardised to unit variance before analyses and ABS was square root transformed before analysis. 0^B indicates that the parameter estimate is bound at 0. Bold values are significant different from 0 (P < 0.05). *NA* = term not applicable. The significance of the heritability and the proportion of phenotypic variance explained by maternal and permanent environment effects is based on the significance of the corresponding variance term in the model.

Table S2 Phenotypic variance-covariance matrix for (standardized) male and female life history traits. Variances are on the diagonal, covariances below the diagonal and correlations above the diagonal (±1SE). These models include all fixed effects detailed in the methods and so the phenotypic variances are conditional on these fixed effects and do not equal one. Phenotypic variances for annual breeding success (ABS) are the sum of residual and permanent environment variances. Phenotypic covariances between survival to breeding age (SBA) and all other traits (which are necessarily expressed only in individuals with SBA=1) are not estimable. These parameters estimates are the values used to estimate selection gradients from selection differentials for each sex separately. Age at first reproduction (AFR) is multiplied by -1 to make any trade-offs negative in sign. L = Longevity.

	AFR	L	ABS
**Females**			
AFR	**0.876±0.077**	0.103±0.062*	**0.231±0.032**
L	0.0954±0.0582*	**0.974±0.080**	**0.105±0.039**
ABS	**0.195±0.032**	**0.0939±0.0356**	**0.817±0.029**
**Males**	AFR	L	ABS
AFR	**0.915±0.151**	**0.347±0.113**	**0.181±0.047**
L	**0.361±0.135**	**1.177±0.150**	**0.133±0.044**
ABS	**0.163±0.049**	**0.136±0.048**	**0.886±0.043**

Bold values are significantly different from 0 (P < 0.05) based on log-likelihood ratio tests of models with the parameter estimated versus fixed to zero in ASReml. *P = 0.07

Table S3 Model showing variances (diagonal), covariances (below diagonal) and correlations (above diagonal) (±1SE) for the minimal models of life history traits in both sexes. Genetic parameters do not have associated significances or standard errors as they are calculated from a fourth order FA model (see Methods). Underlined values highlight between-sex covariances and correlations. Non-genetic matrices were estimated as variance-correlation matrices and thus there are no standard errors on covariances. The permanent environment/residual section of the table presents permanent environment (PE) variances in the upper row and residual variances in the lower row. AFR is multiplied by -1 to make any trade-offs negative in sign.

**Genetic**	Female SBA	Female AFR	Female L	Female ABS	Male SBA	Male AFR	Male L	Male ABS
Female SBA	0.187	0.294	0.00844	-0.221	0.516	-0.138	-0.0639	0.0927
Female AFR	0.0521	0.167	-0.368	0.829	0.408	-0.0658	-0.350	-0.0668
Female L	0.00108	-0.0443	0.0868	-0.510	0.419	0.844	0.622	0.925
Female ABS	-0.0206	0.0730	-0.0323	0.0464	0.180	-0.226	-0.220	-0.21
Male SBA	0.0572	0.0426	0.0316	0.00993	0.0655	0.197	0.625	0.703
Male AFR	-0.0482	-0.0216	0.200	-0.0392	0.0405	0.648	0.190	0.765
Male L	-0.0118	-0.0608	0.0781	-0.0202	0.0682	0.0650	0.181	0.722
Male ABS	0.0101	-0.00684	0.0683	-0.0113	0.0451	0.154	0.0772	0.0629

**Permanent env/ Residual**	Female SBA	Female AFR	Female L	Female ABS		Male SBA	Male AFR	Male L	Male ABS
Female SBA	X	*NA*	*NA*	*NA*	Male SBA	*X*	*NA*	*NA*	*NA*
	**0.637±0.009**	*NA*	*NA*	*NA*		**0.707±0.047**	*NA*	*NA*	*NA*
Female AFR	*NA*	**0.625±0.080**	0.188±0.099*	0.999^{NE}*	Male AFR	*NA*	**0.456±0.208**	0.315±0.235*	0.287±0.212*
	NA	*X*	*NA*	*NA*		*NA*	*X*	*NA*	*NA*
Female L	*NA*	0.137	**0.849±0.089**	**0.484±0.172***	Male L	*NA*	0.19	**0.804±0.145**	0.192±0.147*
	NA	*NA*	*X*	*NA*		*NA*	*NA*	*X*	*NA*
Female ABS	*NA*	0.164	**0.0924**	**0.0429±0.0115**	Male ABS	*NA*	0.0723	0.0647	**0.140±0.030**
	NA	*NA*	*NA*	**0.721±0.017**		*NA*	*NA*	*NA*	**0.648±0.023**

**Maternal**	Female SBA	Female AFR	Male SBA
Female SBA	**0.0740±0.0330**	-0.0359±0.326	**0.835±0.362**
Female AFR	-0.00313	**0.103±0.042**	0.285±0.328
Male SBA	**0.0542**	0.0218	**0.0570±0.0282**

**Year of birth**	Female SBA	Female AFR	Male SBA
Female SBA	**0.0729±0.0252**	0.353±0.250	**0.956±0.083**
Female AFR	0.0263	**0.0765±0.0312**	0.386±0.235
Male SBA	**0.0808**	0.0334	**0.0980±0.0314**

**Year of measurement**	Female SBA

Female SBA	0.0291

Bold values are significantly different from 0 ($P < 0.05$), *X* term not fitted: see methods for details. *NA* covariance or correlation not applicable. [NE]Standard errors not estimable. *covariance is between PE for ABS and residual for other traits (see Methods).

Table S4 FA models of a) female, b) male and c) both-sex G-matrices. The base model contains all fixed effects detailed in the methods, any significant non-genetic random effects (see Table S1) and their associated covariances. Subsequent models describe the log-likelihood of sequential addition of genetic factors (i.e. increasing numbers of elements of **Λ,** with ***Ψ*** fixed at zero; see Methods). Significance of additional factors is assessed by comparing the change in log-likelihood between models, assuming twice the difference in log-likelihood is χ^2 distributed with the number of degrees of freedom equal to the difference in the number of parameters between the models (Δdf). Models highlighted in italics are the statistically best supported models. % variance is the total genetic variance in any given model divided by the best estimate of the total variance in the **G-**matrix under consideration: for $\mathbf{G_f}$**,** this is the total variance in the fourth order FA model; for $\mathbf{G_m}$, this is the sum of the univariate estimates of additive genetic variance for each trait; and for the both-sex model, this is the sum of these two values.

a) FA models of $\mathbf{G_f}$				
Number of factors	Log likelihood	Δdf	P-value	% variance
Base	-2365.75			
1	-2352.07	4	<0.001	47%
2	*-2348.14*	*3*	*0.0490*	*90%*
3	-2348.03	2	0.896	93%
4	-2348.01	1	0.841	100%

b) FA models of $\mathbf{G_m}$				
Base	-1462.10			
1	*-1449.49*	*4*	*<0.001*	*50%*
2	-1446.25	3	0.090	114%
3	no convergence			

c) FA models of $\mathbf{G_{bs}}$				
Base	-3813.39			
1	-3790.77	8	<0.001	43%
2	*-3782.36*	*7*	*0.0186*	*55%*
3	-3776.89	6	0.090	85%
4	-3773.51	5	0.239	118%
5	no convergence			

Table S5 Female (co)variance components. Within-sex variances (diagonal) covariances (lower off diagonal) and correlations (upper off diagonal) between female life history traits (±1SE) from a multivariate model of all female traits simultaneously. Genetic parameters do not have associated significances or standard errors as they are calculated from a fourth order FA model where significance values and errors are given on factor estimates, not on the subsequently recovered $\hat{\mathbf{G}}$ (see Table S8 for the non-FA estimate of $\mathbf{G_f}$ with associated errors on each element). Non-genetic matrices were estimated as variance-correlation matrices as these models proved more stable than unstructured variance-covariance models (Gilmour *et al.* 2009) and thus there are no standard errors on covariances. The permanent env/residual section of the table presents permanent environment (PE) variances in the upper row and residual variances in the lower row. AFR is multiplied by -1 to make any trade-offs negative in sign. Variances are presented for comparison with univariate models.

	SBA	AFR	L	ABS
**Genetic**				
SBA	0.165	0.220	0.147	-0.300
AFR	0.0360	0.163	-0.574	0.787
L	0.0161	-0.0624	0.0727	-0.696
ABS	-0.0257	0.0669	-0.0396	0.0444
**Permanent env/ Residual**				
SBA	*X*	*NA*	*NA*	*NA*
	0.660±0.057	*NA*	*NA*	*NA*
AFR	*NA*	**0.624±0.083**	0.206±.0110*	**0.999**^{NE}*****
	NA	*X*	*NA*	*NA*
L	*NA*	0.152	**0.867±0.121**	**0.504±0.172***
	NA	*NA*	*X*	*NA*
ABS	*NA*	**0.168**	**0.0997**	**0.0452±0.0123**
	NA	*NA*	*NA*	**0.720±0.0171**
**Birth year**				
SBA	**0.0634±0.0228**	0.381±0.249	*NA*	*NA*
AFR	0.0270	**0.0792±0.0322**	*NA*	*NA*
L	*NA*	*NA*	*X*	*NA*
ABS	*NA*	*NA*	*NA*	*X*
**Maternal**				
SBA	**0.0689±0.0333**	0.0145±0.360	*NA*	*NA*
AFR	0.0123	**0.105±0.043**	*NA*	*NA*
L	*NA*	*NA*	*X*	*NA*
ABS	*NA*	*NA*	*NA*	*X*
**Year of measurement**				
SBA	*X*	*NA*	*NA*	*NA*
AFR	*NA*	*X*	*NA*	*NA*
L	*NA*	*NA*	*X*	*NA*
ABS	*NA*	*NA*	*NA*	**0.0293±0.0090**

Bold values are significantly different from 0 ($P < 0.05$). *X* term not fit, see methods for details. *NA* covariance or correlation not applicable. *covariance is between PE for ABS and residual for other traits, estimated by forcing residual variance into permanent environment variance as detailed in the methods. ^{NE}Standard errors not estimable.

Table S6 Male (co)variance components. Within-sex variances (diagonal), covariances (lower off diagonal) and correlations (upper off diagonal) between male life history traits (±1SE) from a multivariate model of all male traits simultaneously. Genetic parameters do not have associated significances or standard errors as they are calculated from a second order FA model where significance values and errors are given on factor estimates not on the subsequently recovered **G**-matrix. Non-genetic matrices were estimated as variance-correlation matrices as these models proved more stable than unstructured variance-covariance models (Gilmour *et al.* 2009) and thus there are no standard errors on covariances. The permanent env/residual section of the table presents permanent environment (PE) variances in the upper row and residual variances in the lower row. AFR is multiplied by -1 to make any trade-offs negative in sign. Variances are presented for comparison with estimates from univariate models, but year of measurement variance was not significant for any trait and was thus not fit in the multivariate model of male traits.

	SBA	AFR	L	ABS
**Genetic**				
SBA	0.0386	0.110	1.00	0.708
AFR	0.0172	0.626	0.104	0.781
L	0.0774	0.0323	0.155	0.703
ABS	0.0380	0.167	0.0756	0.0746
**Permanent env/ Residual**				
SBA	*X*	*NA*	*NA*	*NA*
	**0.727±0.045**	*NA*	*NA*	*NA*
AFR	*NA*	**0.471±0.212**	0.339±0.232*	0.238±0.225*
	NA	*X*	*NA*	*NA*
L	*NA*	0.221	**0.829±0.135**	0.204±0.156*
	NA	*NA*	*X*	*NA*
ABS	*NA*	0.0508	0.0490	**0.129±0.032**
	NA	*NA*	*NA*	**0.649±0.023**
**Birth year**				
SBA	**0.0846±0.0279**	*NA*	*NA*	*NA*
AFR	*NA*	*X*	*NA*	*NA*
L	*NA*	*NA*	*X*	*NA*
ABS	*NA*	*NA*	*NA*	*X*
**Maternal**				
SBA	**0.0624±0.0282**	*NA*	*NA*	*NA*
AFR	*NA*	*X*	*NA*	*NA*
L	*NA*	*NA*	*X*	*NA*
ABS	*NA*	*NA*	*NA*	*X*

Bold values are significantly different from 0 ($P < 0.05$), *X* term not fit see methods for detials. *NA* covariance or correlation not applicable. *covariance is between PE for ABS and residual for other traits, estimated by forcing residual variance into permanent environment variance as detailed in the methods.

Table S7 Principal components analysis (PCA) of $\hat{\mathbf{G}}$ estimated from the maximal FA model possible for a) female and b) male and c) both-sex genetic variance-covariance matrices. Eigenvalues indicate the variance explained by each eigenvector and the eigenvectors indicate the loadings of each trait onto each eigenvalue. The number of axes of variation (non-zero eigenvalues) is limited by the number of factors describing $\hat{\mathbf{G}}$ (i.e. four for females and both-sexes and two for males (SI Table S4, above)). PC decomposition of reduced rank **G** was achieved by converting the elements of $\mathbf{\Lambda}$ into $\hat{\mathbf{G}}$ using equation (3) and then running a PC analysis on $\hat{\mathbf{G}}$. fSBA refers to female SBA, mSBA to male SBA etc..

a) PCA of fourth order FA estimate of $\mathbf{G_f}$

	PC1	PC2	PC3	PC4
Eigenvalues	0.230	0.176	0.0338	0.00446
% variance	51.8	39.6	7.6	1.0
Eigenvectors				
fSBA	0.220	0.930	-0.202	0.216
fAFR	0.823	-0.0150	0.423	-0.379
fL	-0.391	0.255	0.876	0.119
fABS	0.349	-0.265	0.112	0.892

b) PCA of second order FA estimate of $\mathbf{G_m}$

	PC1	PC2
Eigenvalues	0.680	0.214
% variance	76.1	23.9
Eigenvectors		
mSBA	0.0551	-0.413
mAFR	0.952	0.209
mL	0.107	-0.830
mABS	0.281	-0.310

c) PCA of fourth order FA estimate of $\mathbf{G_{bs}}$

	PC1	PC2	PC3	PC4
Eigenvalues	0.787	0.267	0.248	0.143
% variance	54.5	18.5	17.1	9.9
Eigenvectors				
fSBA	-0.0668	0.483	-0.531	0.601
fAFR	-0.0826	0.705	0.0376	-0.445
fL	0.310	-0.0988	-0.178	0.0718
fABS	-0.0744	0.207	0.0988	-0.444
mSBA	0.0851	0.203	-0.412	-0.217
mAFR	0.886	0.206	0.269	0.0828
mL	0.188	-0.367	0.614	-0.411
mABS	0.244	0.0188	-0.235	-0.121

Table S8 Female genetic (co)variance components from a non-factor analytic multivariate model of all female traits simultaneously. Genetic variances are presented on the diagonal, covariances on the lower off diagonal and correlations on the upper off diagonal (±1SE). Non-genetic matrices were identical to those presented in Table S5 and so are not presented here. The parameter estimates for this **G**-matrix are identical to those from the factor analytic model presented in the main manuscript (as expected) and this model is presented to provide estimates of errors for the elements of $\mathbf{G_f}$. Equivalent non-factor analytic multivariate models would not run for $\mathbf{G_m}$ or $\mathbf{G_{bs}}$.

	SBA	AFR	L	ABS
**Genetic**				
SBA	**0.165±0.062**	0.220±0.269	0.147±0.493	-0.300±0.222
AFR	0.0360±0.0450	**0.163±0.083**	-0.574±0.842	**0.787±0.170**
L	0.0161±0.0519	-0.0624±0.0734	0.0727±0.107	-0.696±0.783
ABS	-0.0257±0.0195	**0.0669±0.0294**	-0.0396±0.0311	**0.0444±0.0141**
