# Supplementary material for: A Multivariate Analysis of Genetic Constraints to Life History Evolution in a Wild Population of Red Deer
Source: Genetics. 2014 Oct 2;198(4):1735–49. doi: 10.1534/genetics.114.164319 (PMC4256783; doi:10.1534/genetics.114.164319)
Supplement: Supporting Information [file 659807d2f23709cd3f868f1977c1b272_genetics.114.164319-9.pdf]

File S1

Methods and Discussion

Fixed effects: Fixed effects known to be important in this system were included in models of each of the different life history traits, as follows:

For Survival to Breeding Age (SBA), we included linear and quadratic effects of the mother's age (Coulson *et al.* 2003), mother's population sub-area in the offspring's first two years of life (to account for variation in habitat quality between four different sub-areas of the study site (Coulson *et al.* 1997)) and mother's recent reproductive history (whether or not the female had given birth to a calf the previous year and whether it had survived its first year, five different levels; Naive (N), female had not bred previously; True yeld (TY), female had bred previously but did not breed in the previous year; Summer yeld (SY), female bred in the previous year but the calf died before 1 October; Winter yeld (WY), female bred in the previous year but the calf died between 1 October and 1 May; Milk (M), the female successfully reared a calf in the previous year, for details see (Clutton-Brock *et al.* 1983)).

For Age at First Reproduction (AFR), in females we included an individual's mother's population sub-area in her first two years of life (to account for early life differences in habitat quality, four levels as for SBA). For males this fixed effect was not significant and was thus removed.

For Longevity (L), we included a female's lifetime population sub-area as the area in which she spent most years of her life, whereas for males such information was not available for a large number of individuals and so no fixed effects were included.

Finally, for Annual Breeding Succcess (ABS), for females, we included the fixed effects of a female's age, its quadratic, and recent reproductive history as defined for SBA. For male ABS, age and its quadratic were fitted as fixed effects.

Factor Analytic modelling: Estimating a multivariate **G**-matrix can be difficult because of the number of parameters to be estimated (Kirkpatrick and Meyer 2004; Meyer and Kirkpatrick 2005), a problem which may be exacerbated when using the incomplete pedigrees and modest sample sizes typical of data from natural populations. In an attempt to overcome these issues, we used factor analytic modeling techniques (FA) (Wright 1932; Thompson *et al.* 2003; Kirkpatrick and Meyer 2004; Meyer and Kirkpatrick 2005) to provide a (reduced rank) multivariate estimate of genetic variance-covariance matrixes, considering first either sex separately and then all eight traits across both sexes jointly. FA allows the estimation of the major independent axes of genetic variance in the traits, with each successive axis explaining decreasing variance in **G** allowing a "building-up" approach to modeling **G**: increasing numbers of genetic factors are fitted until either the fitting of additional factors is no longer possible or the model is "full rank" and contains as many genetic factors as traits (see below). By taking a FA approach we can estimate the maximal amount of variation in **G** possible given the constraints of the data.

FA involves modeling the genetic variance-covariance matrix (**G**) as a product of a number *m* of independent linear combinations of the original (*p*) traits such that:

$$\hat{\mathbf{G}} = \mathbf{\Lambda}\mathbf{\Lambda}^{\mathrm{T}} + \boldsymbol{\Psi} \quad (2)$$

where $\hat{\mathbf{G}}$ = a (potentially reduced-rank) estimate of **G**, $\mathbf{\Lambda}$ is a lower triangle matrix of constants that represent loadings of each trait on each factor, ^{T} is the transpose of a matrix and $\boldsymbol{\Psi}$ is a vector of specific variances (Meyer and Kirkpatrick 2008). Factor analysis

becomes similar to a principal components analysis (PCA) when $\boldsymbol{\Psi}$ are fixed to zero such that:

$$\hat{\mathbf{G}} = \mathbf{\Lambda\Lambda}^{\mathbf{T}} \tag{3}$$

Both forms of FA can be performed in ASReml (Thompson *et al.* 2003; Gilmour *et al.* 2009) and the significance of additional factors can be assessed by comparing the log-likelihoods of models with sequentially more (or fewer) factors. The number of degrees of freedom for each model is given by $m(2p\text{-}m+1)/2$ in which p and m are the number of traits and factors respectively. Significance is assessed from twice the difference between the log-likelihoods of successive models, assumed to be chi-squared distributed with degrees of freedom (df) equal to the change in df between models. A full rank FA model, with $\mathbf{\Lambda}$ representing a lower triangle of a matrix of dimension p (for equation (3)), is equivalent to a standard multivariate model of **G**.

Although the majority of previous approaches using FA have focused on assessing the rank of **G** (e.g. Mezey and Houle 2005; Hine and Blows 2006; Mcguigan and Blows 2007; Schroderus *et al.* 2010), it has been demonstrated that sampling variance results in an underestimate of the contribution of the smallest and an overestimate of the contribution of the largest "factor" (or eigenvector), and thus an underestimate of the rank of **G** (Hill and Thompson 1978; Meyer and Kirkpatrick 2008); which is particularly apparent for traits with lower heritability (Hine and Blows 2006). We note also that the number of factors with statistical support will depend on the statistical power of the dataset, and thus that a smaller sample size is likely to result in a conclusion that **G** is of lower rank than with a larger sample size. To avoid these issues we took an alternative approach of "building-up" an FA model, adding additional factors until either **G** was full rank (rank $\mathbf{\Lambda}$ = p (four (within-sex models) or eight (both-sex models) in this case)) or models including

additional factors were not possible (due to failure of convergence). FA allows estimation of $\hat{\mathbf{G}}$ (i.e. $\mathbf{\Lambda\Lambda}^{\mathrm{T}}$) that contains the maximum possible variance estimable given the data and thus the best possible estimate of **G** to subsequently assess its potential to generate evolutionary constraint (see below). Because the leading factors to be estimated are those that contain the most variance, any unestimable factors in our analysis should explain considerably less variance than those that are estimable and should thus have a much smaller effect on the response to selection than those that are included.

Standard genetic parameter estimates (variances and covariances of the traits) derived from FA models (using equation 3) do not have associated standard errors as the errors estimated are associated with the elements of the factors (i.e. elements of $\mathbf{\Lambda}$) rather than the elements of the recovered $\hat{\mathbf{G}}$. A principal components analysis (PCA) of $\hat{\mathbf{G}}$ (effectively **G** if analyses are full rank) allows presentation of the results of FA models in the more familiar format of eigenvalues and eigenvectors (Schroderus *et al.* 2010).

To assess the informativeness of FA models, where possible we estimated the proportion of total genetic variation explained by different models. Assessing the proportion of genetic variation explained requires deciding on a "best estimate" of the total variance in the traits. Where full rank FA models can be estimated, this was simply the trace of the estimated **G** (i.e. the sum of the genetic variances). Where full rank FA models were not possible, we used the sum of the univariate estimates of the genetic variances. Thus for females the trace of the full rank estimate of $\mathbf{G_f}$ was used, whereas for males, where a full rank model of $\mathbf{G_m}$ would not converge (see below), the sum of the univariate estimates of the genetic variances was used. For $\mathbf{G_{bs}}$ we summed our "best estimates" of the variance in $\mathbf{G_f}$ and $\mathbf{G_m}$. When covariance exists between traits, information about the variance in one

trait can be used to inform estimates of variance in other traits. As such, multivariate models may provide better estimates of the variance in a trait than univariate models and thus it is possible for even reduced rank FA models to explain more variance in **G**, and equally for full rank FA models to explain less variance in **G**, than the sum of the variances obtained from univariate models.

DISCUSSION

Comparison with other results from the Rum red deer population

Three other studies have considered the role of genetic covariances between traits and the prevalence of evolutionary constraints in the Rum red deer study population (Foerster *et al.* 2007; Morrissey *et al.* 2012; Kruuk *et al.* 2014). The overall pattern of negative genetic covariances between female survival and reproductive traits is very similar to that of a previous study on the same population (Morrissey *et al.* 2012). However, there is a difference in the evolvability ratios of female traits between these two studies (R_e = 0.63 in (Morrissey *et al.* 2012) versus 1.06 here). Furthermore, the current study provides little evidence for genetic constraint acting through between sex genetic covariances, whilst a previous study (Foerster *et al.* 2007) reported a strong negative genetic correlation between an estimate of male and female fitness. One major difference between these two previous studies and the current study is in the treatment of early life survival. Here, early life survival is modelled as a trait of the individual and describes survival to three years of age, whereas both previous studies (Foerster *et al.* 2007; Morrissey *et al.* 2012) modelled early life survival only to one year of age and considered it as a trait of the mother. If this trait is removed from the current study, female R_e values are remarkably similar to those of (Morrissey *et al.* 2012) (R_{e_f} = 0.68 in this study (data not shown) vs. 0.63 in (Morrissey *et al.* 2012)) – an observation that illustrates the changes in conclusions that

may arise dependent on exactly which traits are included in an analysis, and exactly how those traits are defined. Ideally, early life survival would be modelled as a trait of the individual with maternal and maternal genetic effects included to allow the estimation of maternal and direct genetic effects and their genetic covariance. However, in the current multivariate analysis this was not possible due to the complexity of the models that would be required. The differences between these studies points to parent-offspring patterns/processes being a potential key area for future study of genetic constraints in this population.

Finally, a multivariate study of sexual selection in relation to antler trait morphology in this population (Kruuk *et al.* 2014) found evidence of genetic variance underlying antler traits and also (as here) male annual breeding success, but – in a test of the potential for antler traits to respond to selection (Morrissey *et al.* 2010) – no evidence of genetic covariances between antler size or shape and the fitness measure. There was also a moderate discrepancy between the direction of maximum genetic variance (gmax) and that of the selection gradients, $\boldsymbol{\beta}$, with a posterior mode of the angle between the two vectors of 37.62° (95%CI 6.43, 62.34). Thus in relation to male fecundity selection for antler morphology, evolutionary constraints appear to be shaped by patterns of genetic covariances, rather than by the genetic variance of individual traits, but a similar pattern emerges of moderate rather than strong constraints.

REFERENCES

Clutton-Brock, T. H., F. E. Guinness and S. D. Albon, 1983 The costs of reproduction to red deer hinds. Journal of Animal Ecology **52:** 367-383.

Coulson, T., S. Albon, F. Guinness, J. Pemberton and T. Cluttonbrock, 1997 Population substructure, local density, and calf winter survival in red deer (*cervus elaphus*). Ecology **78:** 852-863.

Coulson, T., L. E. B. Kruuk, G. Tavecchia, J. M. Pemberton and T. H. Clutton-Brock, 2003 Estimating selection on neonatal traits in red deer using elasticity path analysis. Evolution **57:** 2879-2892.

Foerster, K., T. Coulson, B. C. Sheldon, J. M. Pemberton, T. H. Clutton-Brock *et al.*, 2007 Sexually antagonistic genetic variation for fitness in red deer. Nature **447:** 1107-1109.

Gilmour, A. R., B. J. Gogel, B. R. Cullis and R. Thompson, 2009 *Asreml user guide release 3.0*. VSN International Ltd, Hemel Hempstead, HP1 1ES, UK, www.vsni.co.uk.

Hill, W. G., and R. Thompson, 1978 Probabilities of non-positive definite between-group or genetic covariance matrices. Biometrics **34:** 429-439.

Hine, E., and M. W. Blows, 2006 Determining the effective dimensionality of the genetic variance-covariance matrix. Genetics **173:** 1135-1144.

Kirkpatrick, M., and K. Meyer, 2004 Direct estimation of genetic principal components: Simplified analysis of complex phenotypes. Genetics **168:** 2295-2306.

Kruuk, L. E. B., T. H. Clutton-Brock and J. M. Pemberton, 2014 Case study: Quantitative genetics and sexual selection of weaponry in a wild ungulate. in *Quantitative genetics of wild populations*. Oxford University Press, Oxford,.

Mcguigan, K., and M. W. Blows, 2007 The phenotypic and genetic covariance structure of drosophlid wings. Evolution **61:** 902-911.

Meyer, K., and M. Kirkpatrick, 2005 Restricted maximum likelihood estimation of genetic principal components and smoothed covariance matrices. Genetics Selection Evolution **37:** 1-30.

Meyer, K., and M. Kirkpatrick, 2008 Perils of parsimony: Properties of reduced-rank estimates of genetic covariance matrices. Genetics **180:** 1153-1166.

Mezey, J. G., and D. Houle, 2005 The dimensionality of genetic variation for wing shape in drosophila melanogaster. Evolution **59:** 1027-1038.

Morrissey, M. B., L. E. B. Kruuk and A. J. Wilson, 2010 The danger of applying the breeder's equation in observational studies of natural populations. Journal of Evolutionary Biology **23:** 2277-2288.

Morrissey, M. B., C. A. Walling, A. J. Wilson, J. M. Pemberton, T. H. Clutton-Brock *et al.*, 2012 Genetic analysis of life-history constraint and evolution in a wild ungulate population. The American Naturalist **179:** E97-E114.

Schroderus, E., I. Jokinen, M. Koivula, E. Koskela, T. Mappes *et al.*, 2010 Intra- and intersexual trade-offs between testosterone and immune system: Implications for sexual and sexually antagonistic selection. The American Naturalist **176:** E90-E97.

Thompson, R., B. Cullis, A. Smith and A. Gilmour, 2003 A sparse implementation of the average information algorithm for factor analytic and reduced rank variance models. Australian & New Zealand Journal of Statistics **45:** 445-459.

Wright, S., 1932 General, group and special size factors. Genetics **17:** 603-619.
