# Supplementary material for: A Multivariate Analysis of Genetic Constraints to Life History Evolution in a Wild Population of Red Deer
Source: Genetics. 2014 Oct 2;198(4):1735–49. doi: 10.1534/genetics.114.164319 (PMC4256783; doi:10.1534/genetics.114.164319)
Supplement: Supporting Information [file 64adfc516ffaa788ca5fe9f6117904c6_genetics.114.164319-6.pdf]

Table S1 Estimates of variance components for female and male life history traits from univariate models. N = sample sizes (Obs = number of observations, indiv = number of individuals), SBA = survival to breeding age, AFR = age at first reproduction, ABS = annual breeding success and L = adult longevity. V_A = additive genetic variation, V_M = maternal variation, V_{PE} = permanent environment variation, V_{BY} = birth year variation, V_{YR} = year of measurement variation and V_R = residual variation. min - results from models with non-significant random effects removed. m^2 and pe^2 are the proportion of phenotypic variance explained by maternal and permanent environment effects respectively. All analyses are based on standard deviation standardised data (i.e. have a variance of 1), but models include fixed effects and so V_A is not identical to heritability (h^2). Heritabilities are presented as narrow sense heritabilities, the ratio of the additive genetic variance (V_A) to phenotypic variance (V_P). Coefficients of variance are presented for all components (except year components) as $CV_X = 100 \times \frac{\sqrt{V_X}}{\overline{X}}$, where x = trait of interest and $\overline{X}$ is the mean.

	N (Obs, indiv)	mean*	SD*	V_A±SE	V_M±SE	V_{PE}±SE	V_{BY}±SE	V_{YR}±SE	V_R±SE	h^2±SE	m^2 or pe^2 ±SE	CV_A	CV_M/ CV_{PE}	CV_R
**FEM**														
SBA	1126	1.07	1	**0.16±0.06**	**0.069±0.033**	NA	**0.064±0.023**	*NA*	**0.67±0.06**	**0.17±0.06**	**0.072±0.035**	37.1	24.6	76.2
AFR	519	11.2	1	**0.17±0.09**	**0.14±0.06**	NA	**0.069±0.033**	*NA*	**0.57±0.09**	**0.18±0.09**	**0.15±0.06**	3.72	3.38	5.05
L	338	2.51	1	0.15±0.12	*NA*	*NA*	0.036±0.031	*NA*	**0.77±0.12**	0.16±0.12	*NA*	15.3	*NA*	34.9
min L		2.51	1	0.099±0.11	*NA*	*NA*	*NA*	*NA*	**0.84±0.12**	0.11±0.12	*NA*	12.6	*NA*	36.6
ABS	3859, 439	1.27	1	**0.044±0.016**	*NA*	**0.028±0.014**	0^B	**0.033±0.01**	**0.73±0.02**	**0.053±0.019**	**0.033±0.017**	16.6	13.1	67.0
min ABS		1.27	1	**0.044±0.016**	*NA*	**0.029±0.015**	*NA*	**0.033±0.010**	**0.73±0.02**	**0.053±0.018**	**0.035±0.017**	16.6	13.3	67.0
**MALES**														
SBA	1114	0.85	1	0.053±0.046	**0.060±0.030**	*NA*	**0.080±0.027**	*NA*	**0.72±0.05**	0.059±0.051	**0.066±0.032**	27.2	28.7	99.6
AFR	149	10.8	1	0.40±0.27	0.11±0.15	*NA*	0.054±0.062	*NA*	**0.46±0.24**	0.39±0.25	0.11±0.15	5.84	3.06	6.27
min AFR		10.8	1	0.48±0.27	*NA*	*NA*	*NA*	*NA*	**0.55±0.23**	0.46±0.24	*NA*	6.39	*NA*	6.85
L	245	3.69	1	0.086±0.153	*NA*	*NA*	0.049±0.042	*NA*	**0.86±0.17**	0.086±0.15	*NA*	7.93	*NA*	25.2
min L		3.69	1	0.17±0.17	*NA*	*NA*	*NA*	*NA*	**0.83±0.18**	0.17±0.17	*NA*	11.2	*NA*	24.7
ABS	2004, 570	0.58	1	**0.070±0.032**	*NA*	**0.12±0.03**	0.0085±0.0085	0.0045±0.0042	**0.65±0.023**	**0.082±0.038**	**0.14±0.04**	45.7	59.5	139
min ABS		0.58	1	**0.079±0.033**	*NA*	**0.12±0.03**	*NA*	*NA*	**0.65±0.023**	**0.093±0.038**	**0.14±0.04**	48.6	59.2	140

* NB all phenotypic data were standardised to unit variance before analyses and ABS was square root transformed before analysis. 0^B indicates that the parameter estimate is bound at 0. Bold values are significant different from 0 ($P < 0.05$). *NA* = term not applicable. The significance of the heritability and the proportion of phenotypic variance explained by maternal and permanent environment effects is based on the significance of the corresponding variance term in the model.
