# Supplementary material for: A Multivariate Analysis of Genetic Constraints to Life History Evolution in a Wild Population of Red Deer
Source: Genetics. 2014 Oct 2;198(4):1735–49. doi: 10.1534/genetics.114.164319 (PMC4256783; doi:10.1534/genetics.114.164319)
Supplement: Supporting Information [file b508d577aae8bd204bf83ad04ca825bf_genetics.114.164319-5.pdf]

Table S2 Phenotypic variance-covariance matrix for (standardized) male and female life history traits. Variances are on the diagonal, covariances below the diagonal and correlations above the diagonal (±1SE). These models include all fixed effects detailed in the methods and so the phenotypic variances are conditional on these fixed effects and do not equal one. Phenotypic variances for annual breeding success (ABS) are the sum of residual and permanent environment variances. Phenotypic covariances between survival to breeding age (SBA) and all other traits (which are necessarily expressed only in individuals with SBA=1) are not estimable. These parameters estimates are the values used to estimate selection gradients from selection differentials for each sex separately. Age at first reproduction (AFR) is multiplied by -1 to make any trade-offs negative in sign. L = Longevity.

	AFR	L	ABS
**Females**			
AFR	**0.876±0.077**	0.103±0.062*	**0.231±0.032**
L	0.0954±0.0582*	**0.974±0.080**	**0.105±0.039**
ABS	**0.195±0.032**	**0.0939±0.0356**	**0.817±0.029**
**Males**	AFR	L	ABS
AFR	**0.915±0.151**	**0.347±0.113**	**0.181±0.047**
L	**0.361±0.135**	**1.177±0.150**	**0.133±0.044**
ABS	**0.163±0.049**	**0.136±0.048**	**0.886±0.043**

Bold values are significantly different from 0 ($P < 0.05$) based on log-likelihood ratio tests of models with the parameter estimated versus fixed to zero in ASReml. *P = 0.07
