# Supplementary material for: A Multivariate Analysis of Genetic Constraints to Life History Evolution in a Wild Population of Red Deer
Source: Genetics. 2014 Oct 2;198(4):1735–49. doi: 10.1534/genetics.114.164319 (PMC4256783; doi:10.1534/genetics.114.164319)
Supplement: Supporting Information [file 0ccc6c8c8bd2a9c54f14e79738466e60_genetics.114.164319-2.pdf]

Table S3 Model showing variances (diagonal), covariances (below diagonal) and correlations (above diagonal) (±1SE) for the minimal models of life history traits in both sexes. Genetic parameters do not have associated significances or standard errors as they are calculated from a fourth order FA model (see Methods). Underlined values highlight between-sex covariances and correlations. Non-genetic matrices were estimated as variance-correlation matrices and thus there are no standard errors on covariances. The permanent environment/residual section of the table presents permanent environment (PE) variances in the upper row and residual variances in the lower row. AFR is multiplied by -1 to make any trade-offs negative in sign.

**Genetic**	Female SBA	Female AFR	Female L	Female ABS	Male SBA	Male AFR	Male L	Male ABS
Female SBA	0.187	0.294	0.00844	-0.221	0.516	-0.138	-0.0639	0.0927
Female AFR	0.0521	0.167	-0.368	0.829	0.408	-0.0658	-0.350	-0.0668
Female L	0.00108	-0.0443	0.0868	-0.510	0.419	0.844	0.622	0.925
Female ABS	-0.0206	0.0730	-0.0323	0.0464	0.180	-0.226	-0.220	-0.21
Male SBA	0.0572	0.0426	0.0316	0.00993	0.0655	0.197	0.625	0.703
Male AFR	-0.0482	-0.0216	0.200	-0.0392	0.0405	0.648	0.190	0.765
Male L	-0.0118	-0.0608	0.0781	-0.0202	0.0682	0.0650	0.181	0.722
Male ABS	0.0101	-0.00684	0.0683	-0.0113	0.0451	0.154	0.0772	0.0629

**Permanent env/ Residual**	Female SBA	Female AFR	Female L	Female ABS		Male SBA	Male AFR	Male L	Male ABS
Female SBA	X	*NA*	*NA*	*NA*	Male SBA	*X*	*NA*	*NA*	*NA*
	**0.637±0.009**	*NA*	*NA*	*NA*		**0.707±0.047**	*NA*	*NA*	*NA*
Female AFR	*NA*	**0.625±0.080**	0.188±0.099*	0.999^{NE}*	Male AFR	*NA*	**0.456±0.208**	0.315±0.235*	0.287±0.212*
	NA	*X*	*NA*	*NA*		*NA*	*X*	*NA*	*NA*
Female L	*NA*	0.137	**0.849±0.089**	**0.484±0.172***	Male L	*NA*	0.19	**0.804±0.145**	0.192±0.147*
	NA	*NA*	*X*	*NA*		*NA*	*NA*	*X*	*NA*
Female ABS	*NA*	0.164	**0.0924**	**0.0429±0.0115**	Male ABS	*NA*	0.0723	0.0647	**0.140±0.030**
	NA	*NA*	*NA*	**0.721±0.017**		*NA*	*NA*	*NA*	**0.648±0.023**

**Maternal**	Female SBA	Female AFR	Male SBA
Female SBA	**0.0740±0.0330**	-0.0359±0.326	**0.835±0.362**
Female AFR	-0.00313	**0.103±0.042**	0.285±0.328
Male SBA	**0.0542**	0.0218	**0.0570±0.0282**

**Year of birth**	Female SBA	Female AFR	Male SBA
Female SBA	**0.0729±0.0252**	0.353±0.250	**0.956±0.083**
Female AFR	0.0263	**0.0765±0.0312**	0.386±0.235
Male SBA	**0.0808**	0.0334	**0.0980±0.0314**

**Year of measurement**	Female SBA

Female SBA	0.0291

Bold values are significantly different from 0 ($P < 0.05$), *X* term not fitted: see methods for details. *NA* covariance or correlation not applicable. ^{NE}Standard errors not estimable. *covariance is between PE for ABS and residual for other traits (see Methods).
