# Supplementary material for: A Multivariate Analysis of Genetic Constraints to Life History Evolution in a Wild Population of Red Deer
Source: Genetics. 2014 Oct 2;198(4):1735–49. doi: 10.1534/genetics.114.164319 (PMC4256783; doi:10.1534/genetics.114.164319)
Supplement: Supporting Information [file f52f1099324af3c428257000d6a4c7d4_genetics.114.164319-8.pdf]

Table S4 FA models of a) female, b) male and c) both-sex G-matrices. The base model contains all fixed effects detailed in the methods, any significant non-genetic random effects (see Table S1) and their associated covariances. Subsequent models describe the log-likelihood of sequential addition of genetic factors (i.e. increasing numbers of elements of **Λ,** with ***Ψ*** fixed at zero; see Methods). Significance of additional factors is assessed by comparing the change in log-likelihood between models, assuming twice the difference in log-likelihood is χ^2 distributed with the number of degrees of freedom equal to the difference in the number of parameters between the models (Δdf). Models highlighted in italics are the statistically best supported models. % variance is the total genetic variance in any given model divided by the best estimate of the total variance in the **G-**matrix under consideration: for $\mathbf{G_f}$**,** this is the total variance in the fourth order FA model; for $\mathbf{G_m}$, this is the sum of the univariate estimates of additive genetic variance for each trait; and for the both-sex model, this is the sum of these two values.

a) FA models of $\mathbf{G_f}$				
Number of factors	Log likelihood	Δdf	P-value	% variance
Base	-2365.75			
1	-2352.07	4	<0.001	47%
2	*-2348.14*	*3*	*0.0490*	*90%*
3	-2348.03	2	0.896	93%
4	-2348.01	1	0.841	100%

b) FA models of $\mathbf{G_m}$				
Base	-1462.10			
1	*-1449.49*	*4*	*<0.001*	*50%*
2	-1446.25	3	0.090	114%
3	no convergence			

c) FA models of $\mathbf{G_{bs}}$				
Base	-3813.39			
1	-3790.77	8	<0.001	43%
2	*-3782.36*	*7*	*0.0186*	*55%*
3	-3776.89	6	0.090	85%
4	-3773.51	5	0.239	118%
5	no convergence			
