# Supplementary material for: A Multivariate Analysis of Genetic Constraints to Life History Evolution in a Wild Population of Red Deer
Source: Genetics. 2014 Oct 2;198(4):1735–49. doi: 10.1534/genetics.114.164319 (PMC4256783; doi:10.1534/genetics.114.164319)
Supplement: Supporting Information [file 97c27c54b81c7ea734ce580d0db74792_genetics.114.164319-7.pdf]

Table S5 Female (co)variance components. Within-sex variances (diagonal) covariances (lower off diagonal) and correlations (upper off diagonal) between female life history traits (±1SE) from a multivariate model of all female traits simultaneously. Genetic parameters do not have associated significances or standard errors as they are calculated from a fourth order FA model where significance values and errors are given on factor estimates, not on the subsequently recovered $\hat{\mathbf{G}}$ (see Table S8 for the non-FA estimate of $\mathbf{G_f}$ with associated errors on each element). Non-genetic matrices were estimated as variance-correlation matrices as these models proved more stable than unstructured variance-covariance models (Gilmour *et al.* 2009) and thus there are no standard errors on covariances. The permanent env/residual section of the table presents permanent environment (PE) variances in the upper row and residual variances in the lower row. AFR is multiplied by -1 to make any trade-offs negative in sign. Variances are presented for comparison with univariate models.

	SBA	AFR	L	ABS
**Genetic**				
SBA	0.165	0.220	0.147	-0.300
AFR	0.0360	0.163	-0.574	0.787
L	0.0161	-0.0624	0.0727	-0.696
ABS	-0.0257	0.0669	-0.0396	0.0444
**Permanent env/ Residual**				
SBA	*X*	*NA*	*NA*	*NA*
	0.660±0.057	*NA*	*NA*	*NA*
AFR	*NA*	**0.624±0.083**	0.206±.0110*	**0.999^{NE}***
	NA	*X*	*NA*	*NA*
L	*NA*	0.152	**0.867±0.121**	**0.504±0.172***
	NA	*NA*	*X*	*NA*
ABS	*NA*	**0.168**	**0.0997**	**0.0452±0.0123**
	NA	*NA*	*NA*	**0.720±0.0171**
**Birth year**				
SBA	**0.0634±0.0228**	0.381±0.249	*NA*	*NA*
AFR	0.0270	**0.0792±0.0322**	*NA*	*NA*
L	*NA*	*NA*	*X*	*NA*
ABS	*NA*	*NA*	*NA*	*X*
**Maternal**				
SBA	**0.0689±0.0333**	0.0145±0.360	*NA*	*NA*
AFR	0.0123	**0.105±0.043**	*NA*	*NA*
L	*NA*	*NA*	*X*	*NA*
ABS	*NA*	*NA*	*NA*	*X*
**Year of measurement**				
SBA	*X*	*NA*	*NA*	*NA*
AFR	*NA*	*X*	*NA*	*NA*
L	*NA*	*NA*	*X*	*NA*
ABS	*NA*	*NA*	*NA*	**0.0293±0.0090**

Bold values are significantly different from 0 ($P < 0.05$). *X* term not fit, see methods for details. *NA* covariance or correlation not applicable. *covariance is between PE for ABS and residual for other traits, estimated by forcing residual variance into permanent environment variance as detailed in the methods. ^{NE}Standard errors not estimable.
