# Supplementary material for: A Multivariate Analysis of Genetic Constraints to Life History Evolution in a Wild Population of Red Deer
Source: Genetics. 2014 Oct 2;198(4):1735–49. doi: 10.1534/genetics.114.164319 (PMC4256783; doi:10.1534/genetics.114.164319)
Supplement: Supporting Information [file 809cc9b4e4d6305751338aa7012991d0_genetics.114.164319-4.pdf]

Table S6 Male (co)variance components. Within-sex variances (diagonal), covariances (lower off diagonal) and correlations (upper off diagonal) between male life history traits (±1SE) from a multivariate model of all male traits simultaneously. Genetic parameters do not have associated significances or standard errors as they are calculated from a second order FA model where significance values and errors are given on factor estimates not on the subsequently recovered **G**-matrix. Non-genetic matrices were estimated as variance-correlation matrices as these models proved more stable than unstructured variance-covariance models (Gilmour *et al.* 2009) and thus there are no standard errors on covariances. The permanent env/residual section of the table presents permanent environment (PE) variances in the upper row and residual variances in the lower row. AFR is multiplied by -1 to make any trade-offs negative in sign. Variances are presented for comparison with estimates from univariate models, but year of measurement variance was not significant for any trait and was thus not fit in the multivariate model of male traits.

	SBA	AFR	L	ABS
**Genetic**				
SBA	0.0386	0.110	1.00	0.708
AFR	0.0172	0.626	0.104	0.781
L	0.0774	0.0323	0.155	0.703
ABS	0.0380	0.167	0.0756	0.0746
**Permanent env/ Residual**				
SBA	*X*	*NA*	*NA*	*NA*
	**0.727±0.045**	*NA*	*NA*	*NA*
AFR	*NA*	**0.471±0.212**	0.339±0.232*	0.238±0.225*
	NA	*X*	*NA*	*NA*
L	*NA*	0.221	**0.829±0.135**	0.204±0.156*
	NA	*NA*	*X*	*NA*
ABS	*NA*	0.0508	0.0490	**0.129±0.032**
	NA	*NA*	*NA*	**0.649±0.023**
**Birth year**				
SBA	**0.0846±0.0279**	*NA*	*NA*	*NA*
AFR	*NA*	*X*	*NA*	*NA*
L	*NA*	*NA*	*X*	*NA*
ABS	*NA*	*NA*	*NA*	*X*
**Maternal**				
SBA	**0.0624±0.0282**	*NA*	*NA*	*NA*
AFR	*NA*	*X*	*NA*	*NA*
L	*NA*	*NA*	*X*	*NA*
ABS	*NA*	*NA*	*NA*	*X*

Bold values are significantly different from 0 ($P < 0.05$), *X* term not fit see methods for detials. *NA* covariance or correlation not applicable. *covariance is between PE for ABS and residual for other traits, estimated by forcing residual variance into permanent environment variance as detailed in the methods.
