# Supplementary material for: A Multivariate Analysis of Genetic Constraints to Life History Evolution in a Wild Population of Red Deer
Source: Genetics. 2014 Oct 2;198(4):1735–49. doi: 10.1534/genetics.114.164319 (PMC4256783; doi:10.1534/genetics.114.164319)
Supplement: Supporting Information [file 9f125227053518bb65bc83804732ddfb_genetics.114.164319-10.pdf]

Table S7 Principal components analysis (PCA) of $\hat{\mathbf{G}}$ estimated from the maximal FA model possible for a) female and b) male and c) both-sex genetic variance-covariance matrices. Eigenvalues indicate the variance explained by each eigenvector and the eigenvectors indicate the loadings of each trait onto each eigenvalue. The number of axes of variation (non-zero eigenvalues) is limited by the number of factors describing $\hat{\mathbf{G}}$ (i.e. four for females and both-sexes and two for males (SI Table S4, above)). PC decomposition of reduced rank **G** was achieved by converting the elements of $\mathbf{\Lambda}$ into $\hat{\mathbf{G}}$ using equation (3) and then running a PC analysis on $\hat{\mathbf{G}}$. fSBA refers to female SBA, mSBA to male SBA etc..

a) PCA of fourth order FA estimate of $\mathbf{G_f}$				
	PC1	PC2	PC3	PC4
Eigenvalues	0.230	0.176	0.0338	0.00446
% variance	51.8	39.6	7.6	1.0
Eigenvectors				
fSBA	0.220	0.930	-0.202	0.216
fAFR	0.823	-0.0150	0.423	-0.379
fL	-0.391	0.255	0.876	0.119
fABS	0.349	-0.265	0.112	0.892
b) PCA of second order FA estimate of $\mathbf{G_m}$				
	PC1	PC2		
Eigenvalues	0.680	0.214		
% variance	76.1	23.9		
Eigenvectors				
mSBA	0.0551	-0.413		
mAFR	0.952	0.209		
mL	0.107	-0.830		
mABS	0.281	-0.310		
c) PCA of fourth order FA estimate of $\mathbf{G_{bs}}$				
	PC1	PC2	PC3	PC4
Eigenvalues	0.787	0.267	0.248	0.143
% variance	54.5	18.5	17.1	9.9
Eigenvectors				
fSBA	-0.0668	0.483	-0.531	0.601
fAFR	-0.0826	0.705	0.0376	-0.445
fL	0.310	-0.0988	-0.178	0.0718
fABS	-0.0744	0.207	0.0988	-0.444
mSBA	0.0851	0.203	-0.412	-0.217
mAFR	0.886	0.206	0.269	0.0828
mL	0.188	-0.367	0.614	-0.411
mABS	0.244	0.0188	-0.235	-0.121
