# Supplementary material for: A Multivariate Analysis of Genetic Constraints to Life History Evolution in a Wild Population of Red Deer
Source: Genetics. 2014 Oct 2;198(4):1735–49. doi: 10.1534/genetics.114.164319 (PMC4256783; doi:10.1534/genetics.114.164319)
Supplement: Supporting Information [file 021e7d58888790c18f6ea1e642e3c78f_genetics.114.164319-3.pdf]

Table S8 Female genetic (co)variance components from a non-factor analytic multivariate model of all female traits simultaneously. Genetic variances are presented on the diagonal, covariances on the lower off diagonal and correlations on the upper off diagonal (±1SE). Non-genetic matrices were identical to those presented in Table S5 and so are not presented here. The parameter estimates for this **G**-matrix are identical to those from the factor analytic model presented in the main manuscript (as expected) and this model is presented to provide estimates of errors for the elements of $\mathbf{G_f}$. Equivalent non-factor analytic multivariate models would not run for $\mathbf{G_m}$ or $\mathbf{G_{bs}}$.

	SBA	AFR	L	ABS
**Genetic**				
SBA	**0.165±0.062**	0.220±0.269	0.147±0.493	-0.300±0.222
AFR	0.0360±0.0450	**0.163±0.083**	-0.574±0.842	**0.787±0.170**
L	0.0161±0.0519	-0.0624±0.0734	0.0727±0.107	-0.696±0.783
ABS	-0.0257±0.0195	**0.0669±0.0294**	-0.0396±0.0311	**0.0444±0.0141**
